# A 725-bp quadruple repeat in the promoter of *SmMYB113* is associated with light-independent anthocyanin regulation in eggplant

**DOI:** 10.1093/hr/uhaf319

**Published:** 2025-11-21

**Authors:** Zhilei Xia, Meng Yang, Yinggemei Huang, Bingxin Yu, Tingxia Wan, Duanhua Wang, Qian Li, Manoj Sapkota, Shuangshuang Yan, Bihao Cao, Zhengkun Qiu

**Affiliations:** College of Horticulture, Key Laboratory of Biology and Genetic Improvement of Horticultural Crops (South China), Ministry of Agriculture and Rural Affairs/Guangdong Vegetable Engineering and Technology Research Center, South China Agricultural University, Guangzhou 510642, China; College of Horticulture, Key Laboratory of Biology and Genetic Improvement of Horticultural Crops (South China), Ministry of Agriculture and Rural Affairs/Guangdong Vegetable Engineering and Technology Research Center, South China Agricultural University, Guangzhou 510642, China; College of Horticulture, Key Laboratory of Biology and Genetic Improvement of Horticultural Crops (South China), Ministry of Agriculture and Rural Affairs/Guangdong Vegetable Engineering and Technology Research Center, South China Agricultural University, Guangzhou 510642, China; College of Horticulture, Key Laboratory of Biology and Genetic Improvement of Horticultural Crops (South China), Ministry of Agriculture and Rural Affairs/Guangdong Vegetable Engineering and Technology Research Center, South China Agricultural University, Guangzhou 510642, China; College of Horticulture, Key Laboratory of Biology and Genetic Improvement of Horticultural Crops (South China), Ministry of Agriculture and Rural Affairs/Guangdong Vegetable Engineering and Technology Research Center, South China Agricultural University, Guangzhou 510642, China; Yuelushan Laboratory, Hunan Vegetable Research Institute, Changsha 410125, China; Yuelushan Laboratory, Hunan Vegetable Research Institute, Changsha 410125, China; Department of Horticulture, University of Kentucky, Lexington, KY 40546, USA; College of Horticulture, Key Laboratory of Biology and Genetic Improvement of Horticultural Crops (South China), Ministry of Agriculture and Rural Affairs/Guangdong Vegetable Engineering and Technology Research Center, South China Agricultural University, Guangzhou 510642, China; College of Horticulture, Key Laboratory of Biology and Genetic Improvement of Horticultural Crops (South China), Ministry of Agriculture and Rural Affairs/Guangdong Vegetable Engineering and Technology Research Center, South China Agricultural University, Guangzhou 510642, China; College of Horticulture, Key Laboratory of Biology and Genetic Improvement of Horticultural Crops (South China), Ministry of Agriculture and Rural Affairs/Guangdong Vegetable Engineering and Technology Research Center, South China Agricultural University, Guangzhou 510642, China

## Abstract

Eggplant exhibits a diverse range of fruit colors, making it an excellent model for studying fruit pigmentation and its genetic regulation. While genes responsible for green and photosensitive purple fruit have been identified, the genetic basis of the nonphotosensitive (NPS) fruit trait in eggplant has remained elusive. In this study, we characterized a major quantitative trait locus (QTL), *SmNPS10.1*, on chromosome 10 using QTL-seq. By combining linkage-based gene mapping with progeny testing, we fine-mapped *SmNPS10.1* to a 33.58-kb interval, within which we identified *SmMYB113*, an R2R3-MYB transcription factor that regulates anthocyanin biosynthesis, as the candidate gene. Sequence analysis identified a unique 725-bp tandem repeat in the *SmMYB113* promoter, present in four copies in NPS eggplant variety 21E27 but only a single copy in photosensitive varieties. This suggests that increased copy number of the repeat may drive light-independent expression of *SmMYB113*. Transgenic complementation confirmed the additional three copies of the 725-bp repeat in the promoter of *SmMYB113* contributes to light-independent anthocyanin regulation. Additionally, we validated the KASP markers 21QP381 (linked to anthocyanin-present fruit color) and 23QP715 (linked to NPS fruit color) across multiple populations, providing powerful tools for marker-assisted selection in eggplant breeding. Our findings offer new insights into the molecular mechanisms controlling fruit color in eggplant and lay the groundwork for the development of molecular markers to facilitate breeding for NPS and other fruit color variants.

## Introduction

Eggplant (*Solanum melongena* L.) is a widely cultivated crop known for its remarkable diversity in fruit colors, ranging from white, green, lilac, and purple to black-purple, which significantly influences its marketability and consumer appeal [1, 2]. The color of eggplant fruit is primarily determined by the presence and accumulation of two key pigments: chlorophyll, responsible for the green color and essential for photosynthesis, and anthocyanins, which contribute to the red, purple, and blue hues of the fruit’s skin [3–6].

As a major factor determining color, anthocyanin biosynthesis and regulation have been well studied in many plants. Anthocyanin biosynthesis in plants is tightly controlled by the MYB-bHLH-WD40 (MBW) complex, among which the MYB transcription factors (TFs), specifically the subgroup 6 (SG6) MYB factors, serve as pivotal regulators in the biosynthesis of anthocyanin pigments [7, 8]. A number of SG6 MYB activators that regulate anthocyanin accumulation have been identified in a variety of plants, including PhAN2 in petunia (*Petunia hybrida*) [8], SlAN2/SlMYB75 and SlAN2-like in tomato (*Solanum lycopersicum*) [9–11], VvMYBA1 and VvMYBA2 in grapes (*Vitis vinifera*) [12], MdMYB10 and MdMYB110a in apples (*Malus domestica*) [13–15], and PpMYB10.1 in peaches (*Prunus persica*) [16]. These MYB TFs typically bind to specific promoter regions of anthocyanin biosynthetic genes, activating their expression and promoting pigment accumulation.

Light is a critical environmental factor influencing anthocyanin production in many plants, with its effect often being dose-dependent [17]. For example, low light levels can result in poor coloration and reducing anthocyanin content, diminishing the visual quality and commercial value of fruits such as apples and tomatoes [18, 19]. In eggplant, the intensity of anthocyanin production is also influenced by light exposure, with some varieties exhibiting light-depende nt (photosensitive) anthocyanin biosynthesis [20, 21]. However, some eggplant varieties exhibit nonphotosensitive (NPS) fruit color, where fruit pigmentation is not affected by light conditions but is instead governed by genetic factors [22]. Additionally, some varieties show reduced photosensitivity, making eggplant an intriguing model for studying pigment regulation and fruit coloration [23, 24].

Recently, *SmMYB113* on chromosome 10 was identified as a key regulator of anthocyanin biosynthesis in photosensitive eggplant varieties [25]. This gene directly binds to the promoters of anthocyanin-related genes (e.g. *SmCytb5*, *SmGST*, *SmMATE*, *SmASAT3*, and *SmF3′5′M*) to activate their expression [26, 27]. Furthermore, the gene responsible for the NPS trait was fine-mapped to a 290-kb region on chromosome 10, with *SmFTSH10* (filamentation temperature-sensitive 10), harboring a C-base deletion in the fourth exon in NPS varieties, being considered a likely candidate gene for this trait [22]. In addition, the *pind* (*purple in the dark*) mutant, which shows less photosensitive anthocyanin biosynthesis, has been mapped to a region on chromosome 10 (7.72 Mb to 11.71 Mb, ‘guiqie1’ genome version); four candidate genes—*EGP21875* (a MYB domain protein, also called *SmMYB113*), *EGP21950* (an unknown protein), *EGP21953*, and *EGP21961* (both CAAX amino-terminal protease family proteins) —were proposed to be associated with the less-photosensitive phenotype in *pind* [23, 24].

In this study, we identified a new locus, *SmNPS10.1*, which governs NPS anthocyanin biosynthesis in eggplant. Through a combination of BSA-seq, map-based cloning, progeny testing, and genetic complementation, we confirmed that *SmMYB113* is the gene underlying *SmNPS10.1*. This work provides valuable insights into the genetic regulation of eggplant fruit color and offers powerful tools for marker-assisted selection (MAS) in eggplant breeding.

## Results

### Genetic analysis of fruit color in NPS eggplant

**Figure 1:**
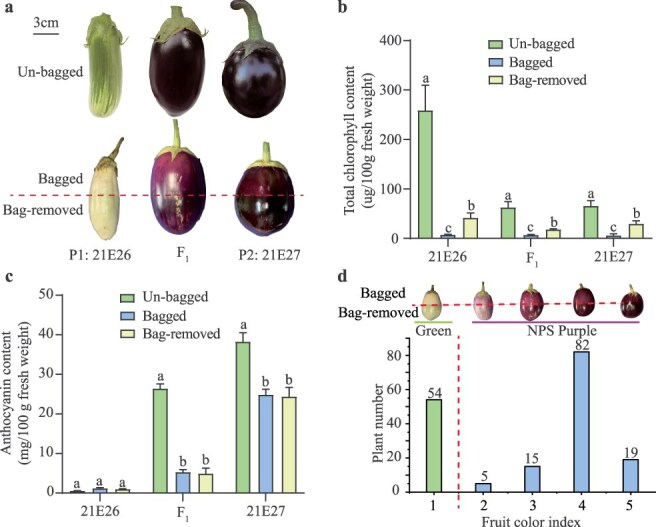
Phenotypic evaluations of green fruit parent 21E26, nonphotosensitive (NPS) fruit parent 21E27, and their derived F_1_, and F_2_ population. (a) Color differences between green fruit parent 21E26, NPS fruit parent 21E27, and their F_1_ generation under un-bagged, bagged, and bag-removed conditions. Total chlorophyll (b) and anthocyanin (c) content in the fruit peels of 21E26, 21E27 and their F_1_ generation under un-bagged, bagged, and bag-removed conditions. Data are means of three biological replicates ± SE. Different letters indicate statistically significant differences among groups (Turkey’s honest significant difference test, *P* < 0.05). (d) Fruit color index frequency histogram in F_2_ population (21E30, *n* = 175).

To investigate the genetic basis of fruit color in NPS eggplant, two parental lines, 21E26 and 21E27, were selected to develop F_1_ and F_2_ populations. Line 21E26 produced green fruits with high chlorophyll content and few anthocyanins in the peel, while both 21E27 and the F_1_ generation bore purple-black fruits with high levels of both chlorophyll and anthocyanins (Fig. 1a-c).

The purple-black coloration in 21E27 fruits was first observed under the sepals four days after pollination (DAP), and this color intensified during fruit development, suggesting that 21E27 is a NPS line (Fig. S1). To confirm this, chlorophyll and anthocyanin contents were measured in both bagged and bag-removed fruit peels. Fruits were bagged for 14 days after pollination, and the bags were then removed from the lower half of the fruit. After an additional seven days, peels from the continuously bagged and bag-removed parts were analyzed. Results showed that chlorophyll levels in the bag-removed portions of 21E26, 21E27, and F_1_ fruits were significantly higher than in the bagged portions (Fig. 1b). However, no significant differences in anthocyanin content were observed between the bagged and nonbagged peel portions in both 21E27 and F_1_ (Fig. 1c), confirming that anthocyanin regulation in these lines occurs in a light-independent manner.

Among 175 individuals in the 21E30 F_2_ population, 121 exhibited nonphotosensitive purple fruit (NPS phenotype) and 54 displayed green fruit (Fig. 1d), consistent with a 3:1 ratio (χ^2^ = 1.8867, *p* = 0.157), suggesting that the NPS trait is controlled by a single dominant gene.

### Identification of candidate loci associated with the NPS trait via QTL-Seq

**Figure 2:**
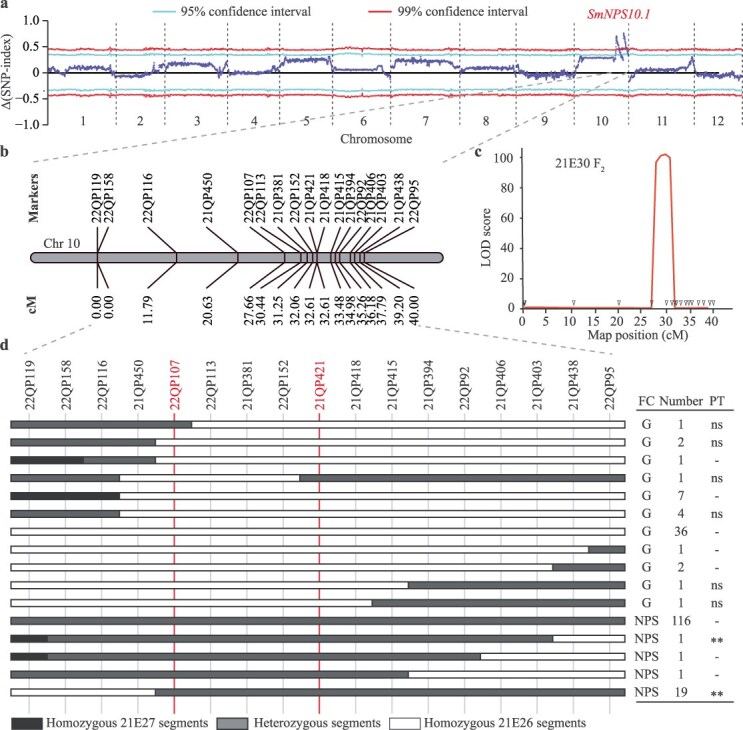
Mapping of NPS fruit color in the 21E30 F_2_ population. (a) The Δ (SNP-index) derived from QTL-seq analysis. The x-axis represents the 12 eggplant chromosomes. The blue line represents the Δ (SNP-index). (b) Linkage map and map distances of the markers used for *SmNPS10.1* mapping. (c) Linkage-based QTL mapping of *SmNPS10.1*. Triangles on the x-axis show the approximate positions of the genotyped markers and their corresponding genetic distances in cM, and the y-axis represents the logarithm of odds (LOD) scores. (d) The genotype and fruit color of the recombinants. FC means fruit color, G means green fruit, NPS means nonphotosensitive fruit, PT means progeny test, ns means no significant differences, ^**^ means *P* < 0.01 (Student’s *t*-test), and - means lines were not selected for progeny test.

*SmFTSH10* was previously considered a potential candidate gene for the NPS trait [22]. However, no mutations, including the C-base deletion, were found in NPS varieties or other fruit-colored eggplants (Fig. S2), suggesting the presence of novel alleles controlling the NPS trait. To identify loci associated with the NPS trait, we performed QTL-seq on 20 F_2_ (21E30) individuals with NPS fruits and 20 with green fruits. This analysis revealed two loci on chromosome 10, spanning from approximately 64.4 Mb to 81.6 Mb, with 99% significance (Fig. 2a and Fig. S3).

To validate these loci, 17 polymorphic KASP markers were developed flanking the two regions (Fig. 2b). Marker-trait association analyses in the entire 21E30 F_2_ population confirmed the presence of a single locus, *SmNPS10.1*, which accounted for 89.33% of the phenotypic variation and exhibited a LOD score of 104.71 (Fig. 2c). *SmNPS10.1* was flanked by markers 22QP107 and 21QP421, spanning a genetic distance of 27.66 cM to 32.61 cM, which corresponded to a physical region from 64 552 099 to 73 818 464 bp on chromosome 10 (Fig. 2c). Progeny testing in F_2:3_ families segregating for *SmNPS10.1* further confirmed this locus, with the *SmNPS10.1* region delimited between the markers 22QP107 and 21QP421, spanning approximately 9.27 Mb (Fig. 2d).

### Fine mapping of *SmNPS10.1*

A total of 106 recombinants were selected from 864 F_2:3_ plants with homozygous 21E27 segments in the *SmNPS10.1* interval (between markers 22QP107 and 21QP421). Marker-trait association mapping narrowed *SmNPS10.1* to a 3.71 cM interval between markers 21QP421 and 21QP450 (Fig. S4). However, the physical distance between the flanking markers increased to approximately 13.6 Mb. When comparing the linear positions of these markers across three published eggplant genomes (SM-V4.1, HQ-1315, and Guiqie1), discrepancies were observed, especially in the SM-V4.1 genome (Fig. S5).

**Figure 3:**
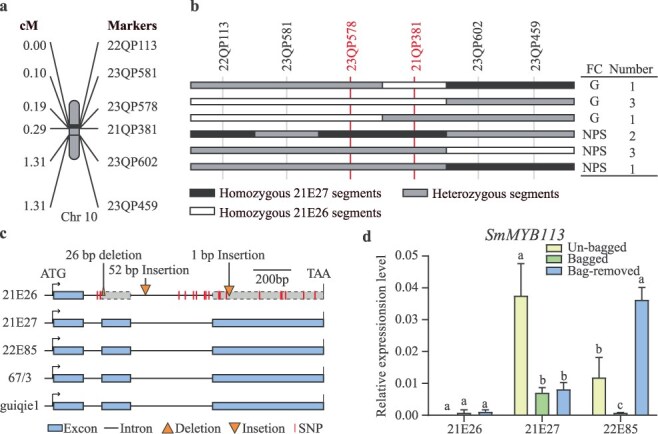
Fine-mapping of the *SmNPS10.1*. (a) Linkage map and map distances of the markers used for *SmNPS10.1* fine-mapping. (b) The genotype and fruit color of the recombinants in the F_5_ populations. FC means fruit color, G means green fruit, and NPS means nonphotosensitive fruit. (c) Mutations detected in the genomic region of the candidate gene (*SmMYB113*) between 21E26 and 21E27. The genomic sequences of *SmMYB113* from photosensitive eggplant varieties 22E85, 67/3, and guiqie1 were used for analysis. (d) Relative expression levels of SmMYB113 in the fruit peels of var. 21E26, 21E27, and 22E85 under un-bagged, bagged, and bag-removed conditions. Relative expression analysis was performed by quantitative reverse transcription (qRT)-PCR. Data are means of three biological replicates ± SE. Different letters indicate statistically significant differences among groups (Tukey’s honest significant difference test, *P* < 0.05).

To refine the mapping, a high-density genetic linkage map with 18 markers surrounding the *SmNPS10.1* region was developed using an 864 F_3:4_ population. Markers 21QP394 and 23QP459 were used to screen recombinants, narrowing the *SmNPS10.1* interval to an 86 kb region (Fig. S6). Further confirmation in 21 F_4:5_ progeny families supported this mapping (Fig. S6). To further narrow down the *SmNPS10.1* mapping region, 11 recombinants between marker 23QP578 and 23QP602 were selected from the 864 F_4:5_ population (Fig. 3a and b). Through marker-trait association analyzing, *SmNPS10.1* was finally mapped in a 33.58-kb region with a genetic distance of 0.1 cM (Fig. 3a and b). The flanking markers were 23QP578 and 21QP381, and the region was validated using a new F_2_ population (24AE009) derived from a green-fruited eggplant (24AE006) and a NPS eggplant (24AE005; Fig. S7).

### *SmMYB113*: The candidate gene for *SmNPS10.1*

Within the *SmNPS10.1* interval, we identified only one gene, *SmMYB113*, which encodes an R2R3-MYB transcription factor previously implicated in regulating anthocyanin biosynthesis in eggplant [27]. Sequence analysis revealed multiple mutations in the *SmMYB113* gene in the NPS line 21E27 compared to 21E26, including a 26-bp deletion (overlapping the first intron and the second exon), 2 insertions (one 52-bp and one 1-bp insertion in the second intron and third exon, respectively), and 18 single nucleotide polymorphisms (SNPs; 9 in introns and 9 in exons; Fig. 3c and Fig. S8). The 26-bp deletion in 21E26 causes alternative splicing, resulting in a truncated protein (Fig. S9).

Expression analysis showed that *SmMYB113* was highly expressed in the tender leaves, flowers and fruit peels (both bagged and nonbagged) in 21E27 (Fig. 3d and Fig. S10). Notably, no significant change in *SmMYB113* expression was observed in the fruit peel before and after bag removal (Fig. 3d), indicating light-independent expression. Based on these results, we conclude that *SmMYB113* is the most likely candidate gene underlying *SmNPS10.1*.

### A 725-bp quadruple repeat unit was identified in the promoter of *SmMYB113* in the NPS eggplant

A previous study has shown that *SmMYB113* positively controls photosensitive anthocyanin biosynthesis in eggplant fruit [25]. As expected, the expression levels of *SmMYB113* were much lower in the bagged fruit peel compared to the bag-removed fruit peel in the photosensitive eggplant 22E85 (Fig. 3d). However, no mutations were detected in *SmMYB113* genome region (ATG to TAA, including exon and intron) between the photosensitive eggplants (22E85, HQ1315 ,and guiqie1) and the NPS eggplant 21E27 (Fig. S8).

**Figure 4:**
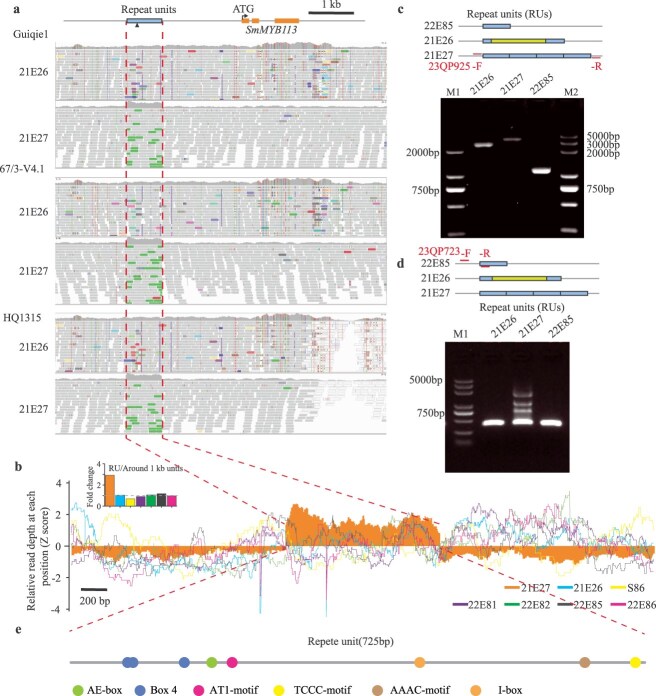
Promoter sequence analysis of *SmMYB113* in eggplant varieties. (a) A high read-enrichment region (RUs, RU) was specifically detected in the NPS line 21E27. Visualizations of read alignments around *SmMYB113* were done using the Integrative Genomics Viewer (IGV). The eggplant genome references of HQ1315, 67/3-V4.1, and Guiqie1 were used for read alignment analysis, respectively. (b) The relative read depth around the RU region. 22E81 bears green fruit, 22E82 and 22E86 bear white fruit, and S86 and 22E85 bear photosensitive purple fruit. (c) PCR analysis shows different sizes of *SmMYB113* promoter region between 21E26, 21E27, and 22E85. (d) PCR analysis shows different copy numbers of RU between 21E26, 21E27, and 22E85. (e) A schematic map of cis-elements in the RU region.

Thus, we investigated the promoter region of *SmMYB113* in NPS and photosensitive eggplants. By visualizing read alignments using the Integrative Genomics Viewer (IGV) with the guiqie1, 67/3 and HQ1315 genome reference, respectively, no mutations were observed except for abnormally high read coverage from −1959 bp to −1234 bp from ATG of *SmMYB113* were detected between 21E27 and the photosensitive varieties guiqie1, 67/3 and HQ1315 (Fig. 4a). The mapped reads depth in this region in 21E27 was ~2.92-fold higher than that of the surrounding 1 kb regions (1 kb upstream and 1 kb downstream from the abnormal region, Fig. 4b). However, this pattern was not found in the photosensitive eggplants, including 21E26 (~1.03-fold), S86 (~0.70-fold), 22E81 (~0.91-fold), 22E82 (~1.08fold), 22E85 (~1.17-fold), and 22E86 (~0.97-fold, Fig. 4b). Primer 23QP925 flanked the abnormal region were then developed to analyze the sequence in this interval. As a result, the primer 23QP925 PCR products in 21E27 were 748 bp longer and 2175 bp longer than that in 21E26 and 22E85, respectively (Fig. 4c). By Sanger sequencing analyses, a 725 bp repeat unit (RU) was identified in 21E27 (Table S1). The RU was present in four copies in 21E27, but only a single copy was found in 21E26 and 22E85 (Fig. 4d). In addition, a 1427-bp insertion was found in 21E26 compared to 21E27 and 22E85.

An additional PCR pair primer 23QP723 (former primer located in the upstream of the RU region, reverse primer located in the RU region), was developed to confirm the RUs in 21E27 and 22E85 (Fig. 4d). Consistent with the sequencing result, four laddery bands (~400 bp, ~1200 bp, ~1800 bp, ~2500 bp) were observed in the PCR produces of 21E27, while only one band was detected in 21E26 and 22E285 (Fig. 4d). Quantitative real-time PCR was further applied to determine the relative copy numbers of the RU in 21E26, 21E27, 22E85, and other 92 eggplant cultivars. As is shown in Fig. S11, the RU copy numbers in NPS eggplants were significantly higher (p = 4.41e^−22^) than that in non-NPS eggplants (white, green, PS, and less PS eggplants).

To explore why *SmMYB113* expressed in a light-independent manner in NPS eggplant fruits, cis-element analysis was conducted using the RU sequences. Among the 51 cis-elements identified in the RU sequences, eight were light responsive motifs, including one AE-box, three box 4, one AT1-motif, one TCCC-motif, one AAAC-motif and one I-box (Fig. 4e and Table S2). In addition, the 725 bp RU sequence has no homologies with sequences in the National Center for Biotechnology Information (NCBI) GenBank and does not have an ORF (Fig. S12). Based on these results, we speculated that the RU repeats in the *SmMYB113* promoter enable light-independent expression of *SmMYB113* in NPS eggplants, thereby enhancing anthocyanin accumulation even in the absence of light.

### *SmMYB113* in NPS fruit regulates anthocyanin biosynthesis in a light-independent manner

**Figure 5:**
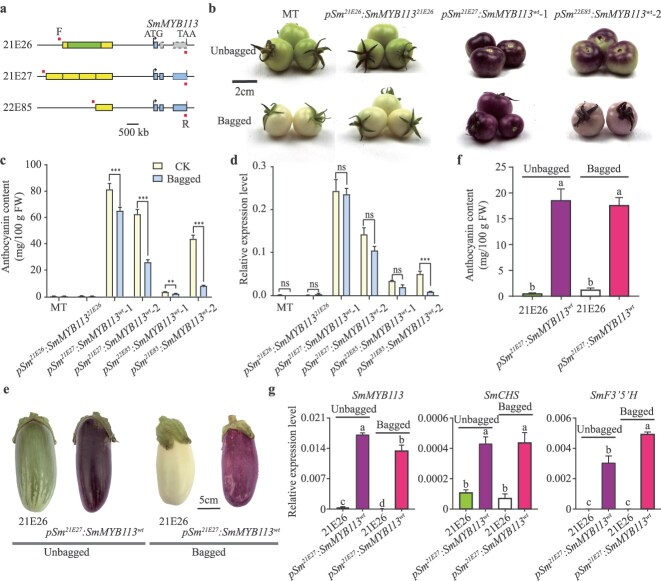
Transgenic assays proved the insertion fragment of the 725 bp RU in the promoter of *SmMYB113* is responsible for nonlight sensitivity. (a) A schematic map of *SmMYB113* genomic sequences with its native promoter used for transgenic work, F and R mean forward and reversed primer that used for transgenic work, respectively. Phenotypic (b), anthocyanin content (c) and relative expression levels of *SmMYB113* (d) analyzed of the tomato fruit of wild type micro-Tom (MT), *proSmMYB113^21E26^:SmMYB113^21E26^* (*pSm^21E26^:SmMYB113^21E26^*), *proSmMYB113^21E27^:SmMYB113^wt^* (*pSm^21E27^:SmMYB113^wt^*), and *proSmMYB113^22E85^:SmMYB113^wt^* (*pSm^22E85^:SmMYB113^wt^*). Phenotypic (e), anthocyanin content (f) and relative expression levels of *SmMYB113* and anthocyanin structural genes (*SmCHS* and *SmF3’5’H*) (g) analyzed in the eggplant fruit of wild type (WT) 21E26, *proSmMYB113^21E26^:SmMYB113^21E26^* (*pSm^21E26^:SmMYB113^21E26^*), *proSmMYB113^21E27^:SmMYB113^wt^* (*pSm^21E27^:SmMYB113^wt^*), and *proSmMYB113^22E85^:SmMYB113^wt^* (*pSm^22E85^:SmMYB113^wt^*). Fruit peels were used for anthocyanin content and gene expression analysis. Relative expression analysis was performed by quantitative reverse transcription (qRT)-PCR. Data are mean of three biological replicates ± SE. ns means no significant difference, ^**^ and ^***^ mean *P* < 0.01 and *P* < 0.001, respectively (Student’s *t*-test). Different letters indicate statistically significant differences among groups (Tukey’s honest significant difference test, *P* < 0.05).

To validate the role of the RU repeat in regulating *SmMYB113* expression, we performed transgenic assays with tomato cultivar micro-Tom (MT) first. The complete *SmMYB113* genomic sequences with its native promoter were amplified from 21E26, 21E27, and 22E85 and inserted into the modified binary vector pCambia2300, respectively (Fig. 5a). Transgenic tomato lines were generated with *SmMYB113* from 21E26 (*proSmMYB113^21E26^:SmMYB113^21E26^*), 21E27 (*proSmMYB113^21E27^:SmMYB113^wt^*), and 22E85 (*proSmMYB113^22E85^:SmMYB113^wt^*), respectively. As a result, anthocyanin accumulation was detected in the unbagged fruits of *proSmMYB113^21E27^*:*SmMYB113^wt^* and *proSmMYB113^22E85^*:*SmMYB113^wt^*, but not in *proSmMYB113^21E26^*:*SmMYB113*^*21E2*6^ (Fig. 5b and c), confirming that the mutations in *SmMYB113* in 21E26 results in a loss of function. In addition, the bagged fruits of *proSmMYB113^21E27^:SmMYB113^wt^* exhibited a darker purple color and higher anthocyanin content compared to the bagged fruits of *proSmMYB113^22E85^:SmMYB113^wt^*, where anthocyanin levels were much lower (Fig. 5b and c). Coincide with the anthocyanin content, *SmMYB113* was highly expressed in the fruit peel of *proSmMYB113^21E27^:SmMYB113^wt^* lines, regardless of whether the fruits were bagged or unbagged (Fig. 5d).

To further investigate the role of the RU in the *SmMYB113* gene of eggplant, we conducted genetic complementation assays. The *SmMYB113* genomic sequence, driven by its native promoter from line 21E27, was introduced into line 21E26. As a result, the transgenic line 21E26 *^proSmMYB11321E27:SmMYB113wt^* produced purple-black fruit with high anthocyanin content, even under light-deficient conditions (Fig. 5c and f). Consistent with this phenotype, the expression levels of *SmMYB113* and anthocyanin biosynthetic genes (*SmCHS* and *SmF3’5’H*) were significantly higher in the fruit peel of the transgenic line compared to the wild-type 21E26. These findings demonstrate that the RU repeat in the *SmMYB113* promoter from 21E27 enables light-independent activation of *SmMYB113*, leading to enhanced anthocyanin accumulation.

### Development and validation of markers for eggplant fruit color

**Figure 6:**
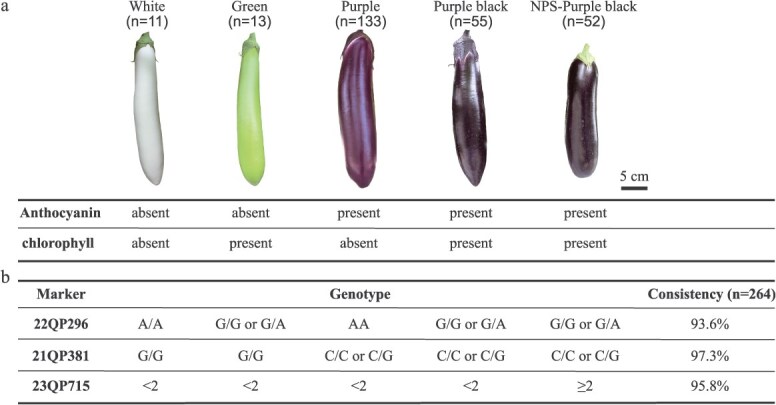
MAS breeding for fruit color of eggplant. (a) The color of eggplant fruit is determined by the presence of anthocyanin and chlorophyll. (b) The genotypes of the developed markers for MAS breeding for eggplant fruit color. The genotype of 23QP715 corresponds to the relative copy number of the RUs in the promoter of *SmMYB113*. *n* = 264 refers to the number of natural cultivars.

Eggplant exhibits a variety of fruit colors, primarily determined by the presence or absence of anthocyanins and chlorophylls (Fig. 6a). To facilitate breeding for specific fruit colors, we developed highly linked markers for these traits. The KASP marker 21QP381, located near *SmMYB113*, was validated in multiple F_2_ and F_3_ populations, showing 100% concordance between genotype and purple fruit phenotype. This marker was also used to screen 264 natural cultivars (including 11 lines bearing white fruit, 13 lines bearing green fruit, 133 lines bearing purple fruit, 55 lines bearing purple-black fruit and 52 lines bearing NPS purple black fruit), yielding a 97.3% consistency rate for purple fruit (including purple, purple-black, and NPS purple-black; Fig. 6b).

Additionally, by screening the same natural cultivars population, marker 23QP715 showed 95.8% consistency for NPS purple-black fruits, while our previously published marker 22QP296 showed 93.6% consistency for chlorophyll-present fruits [28] (Fig. 6b). These results indicated that markers, 22QP296, 21QP381, and 23QP715, can be effectively used for MAS breeding in eggplant.

## Discussion

Our findings identify a key locus on chromosome 10, *SmNPS10.1*, which is associated with the NPS purple-black fruit color. Through a combination of genetic analysis, BSA-seq, and fine mapping, we were able to identify *SmNPS10.1* as the critical locus responsible for the expression of the NPS trait in eggplant. The gene *SmMYB113*, located within this region, emerges as the gene underlying *SmNPS10.1*, offering promising new avenues for marker-assisted breeding to manipulate fruit color in eggplant.

### *SmNPS10.1* as the key locus for NPS fruit color

Our genetic analysis confirmed that the NPS purple fruit color is controlled by a single dominant gene. The F_2_ population segregated in a 3:1 ratio (NPS: green), consistent with Mendelian inheritance patterns for a single dominant gene (Fig. 1d). Notably, the intensity of purple coloration varied among individuals in the F₂ population (Fig. 1d), supporting previous findings that eggplant fruit color is influenced by QTLs [4]. The use of BSA-seq allowed for the identification of two potential loci on chromosome 10, with the locus *SmNPS10.1* being most strongly associated with the NPS phenotype (Fig. 2). These results coincided with a previous study in which several QTLs on chromosome 10 were detected underlying NPS through QTL-seq^22^. Subsequent marker-trait association analyses confirmed *SmNPS10.1* as the locus responsible for nearly 90% of the phenotypic variation in fruit color (Fig. 2). Additionally, the two peaks observed at the end of chromosome 10 in the BSA-seq analysis may be attributed to a large inversion (~12.4 Mb) located near the mapping region, as reported in a recent preprint (https://www.researchsquare.com/article/rs-5083502/v1).

Fine mapping further refined the *SmNPS10.1* locus, identifying a critical interval of approximately 33.58 kb, bounded by markers 23QP578 and 21QP381 (Fig. 3a and b). This fine mapping step provides a much higher resolution for future investigations into the molecular mechanisms underlying the NPS trait. The precise location of *SmNPS10.1* on chromosome 10, along with the development of tightly linked markers such as 21QP381 (Fig. 6), presents a powerful tool for MAS in breeding programs aimed at enhancing or manipulating fruit color in eggplant.

The identification of a unique 725-bp quadruple repeat element in the promoter region of *SmMYB113* in the NPS variety highlights a novel genetic basis for light-independent anthocyanin biosynthesis. Unlike previously characterized mechanisms that rely on light-mediated expression, this repeat element regulates the activation of *SmMYB113*, enabling anthocyanin accumulation irrespective of light exposure. This finding not only establishes *SmNPS10.1* as a previously uncharacterized QTL governing NPS pigmentation but also provides the first evidence of how variations in promoter architecture can modulate light-independent anthocyanin biosynthesis in eggplant. This discovery bridges the gap in understanding the genetic regulation of fruit color diversity and sets the foundation for exploring broader applications in crop improvement. Furthermore, previous studies have demonstrated the impact of mutations and modifications in promoter regions on phenotype regulation [29, 30]. The repeat identified in this study offers an additional avenue to fine-tune anthocyanin biosynthesis, particularly in horticultural crops like eggplant.

The identification of the RU in the promoter region of *SmMYB113* emphasizes the critical role structural variations (SVs) play in regulating complex traits such as light-independent anthocyanin biosynthesis. SVs, including insertions, deletions, and repeat expansions, have been increasingly recognized as key drivers of phenotypic diversity and trait evolution across species. They significantly influence gene expression and protein function, resulting in observable variations in traits such as fruit weight, productivity, and flavor [31–34]. These studies have highlighted how SVs can alter gene expression through changes in promoter architecture, enhancer activity, or alternative splicing. Given the potential of SVs to influence critical traits, systematic studies of genome-wide SVs are essential for identifying novel loci linked to desired phenotypes.

### Role of *SmMYB113* in light-independent anthocyanin regulation

The candidate gene within the *SmNPS10.1* locus, *SmMYB113*, encodes an R2R3-MYB transcription factor that has previously been implicated in the regulation of anthocyanin biosynthesis in eggplant [27]. Our results further validate *SmMYB113* as the key gene controlling anthocyanin accumulation in the NPS fruit (Fig. 5). Sequence analysis revealed several mutations in *SmMYB113* between the NPS cultivar 21E27 and the green cultivar 21E26 (Fig. 3c and Fig. S8). Notably, a 26-bp deletion that overlaps the first intron and exon of *SmMYB113* in 21E26 results in alternative splicing, leading to a truncated protein that is likely nonfunctional (Fig. 3c and Fig. S9). This functional loss in *SmMYB113* explains the lack of anthocyanin accumulation in the green eggplant and the *proSmMYB113^21E26^*:*SmMYB113^21E26^* transgenic plants (Fig. 6). Similar result was also found in the *SmMYB113* in a white eggplant cultivar; a 6-bp deletion was detected in the coding region of the first exon of *SmMYB113* (also named *SmMYB1*), resulting in the lack of two conserved amino acids; this deletion was suggested to disrupt the R2-MYB DNA binding domain and thereby lead to anthocyanin-absent fruit of eggplant [25].

Variations or mutations within the promoter sequences of MYB TFs can lead to altered expression patterns, which in turn affect anthocyanin accumulation. These variations can include small changes such as SNPs, insertions or deletions, as well as larger SVs like repeat expansions. In apple, *MdMYB10* is the key regulator for anthocyanin accumulation in fruit skin. Three genetic variants have been identified in *MdMYB10* promoter region: the first is a 23-bp tandem repeat sequence, providing binding sites for MdMYB10 to auto-regulate its own transcription and leading to red color of fruit skin and flesh; the second is a long terminal repeat retrotransposon insertion, named redTE, located in the upstream of *MdMYB10* and functioned as an enhancer that control the anthocyanin biosynthesis under low light condition. Cultivars failed to effectively accumulate anthocyanin without this enhancer; the third is a transposable element (TE) that influencing *MdMYB10* expression in apple petals [13–15]. In orange, the retrotransposon insertion in the promoter of MYB transcriptional activator *Ruby* increased its expression to confer red fruit flesh [35]. More recently, a LTR retrotransposon located in the promoter of *PsMYB10.2* was identified that may promote its expression and activate the anthocyanin biosynthesis pathway [36].

The *SmMYB113* allele in 21E27, which carries the NPS trait, was highly expressed in both bagged and bag-removed fruit peel, indicating that *SmMYB113^21E27^* regulates anthocyanin biosynthesis in a light-independent manner (Fig. 3d). This light-independent regulation contrasts with the light-dependent expression observed in photosensitive varieties, such as 22E85 (Fig. 3d). However, no mutation was detected in the coding region of *SmMYB113* between 21E27 and the photosensitive varieties (Fig. 3a and Fig. S8). Thus, the identification of the unique 725-bp RU in the promoter of *SmMYB113* in 21E27 suggests that the RU plays a crucial role in driving *SmMYB113* expression without the need for light exposure (Fig. 3). In other words, the presence of RU repeats in the promoter region of *SmMYB113* in NPS eggplants likely upregulates its expression, leading to increased anthocyanin production in a light-independent manner, which is a key characteristic of the NPS trait. This speculation was later confirmed by the genetic complementation assay, which the *proSmMYB113^21E27^*:*SmMYB113^wt^* transgenic plants bear NPS fruit, while the *proSmMYB113^21E26^*:*SmMYB113^wt^* transgenic plants bear photosensitive fruit (Fig. 5). We observed that *proSmMYB113^21E85^:SmMYB113^wt^* transgenic tomato fruit continued to synthesize anthocyanins even after bagging. This may be due to incomplete opacity of the bag seal, allowing light to penetrate and induce anthocyanin biosynthesis. Although we characterized the function of the RU in the promoter of *SmMYB113* in eggplant, additional experiments-such as knocking out or disrupting the tandem RUs in the *SmMYB113* promoter-would provide direct evidence for their role in regulating *SmMYB113* expression and, consequently, in controlling the intensity of purple pigmentation in eggplant fruit. Such functional validation would further clarify the dosage-dependent mechanism underlying light-independent anthocyanin accumulation. Besides, it remains unclear which specific cis-element (s) in the RU or other promoter regions are responsible for regulating *SmMYB113* expression under light-independent conditions. Therefore, identifying the precise regulatory motifs that drive expression in the absence of light will be an important focus of future research.

### Marker development for fruit color breeding

The ability to select for specific fruit color traits, such as NPS eggplant fruit, can not only enhance the aesthetic appeal of eggplant but also provide greater flexibility for breeding varieties that cater to different consumer preferences [2, 6]. The identification of *SmMYB113* as the underlying gene for the NPS fruit color trait has practical implications for eggplant breeding. The KASP marker 21QP381, which is tightly linked to the *SmMYB113* locus, showed perfect genotype–phenotype consistency in various populations, including F_2_, F_2:3_, F_3:4_, and 264 natural cultivars (Fig. 6). This marker offers a reliable tool for identifying anthocyanin-absent or -present eggplants (white/green versus purple/purple-black/NPS) in breeding programs. In addition, the marker 23QP715, which is associated with NPS fruit color, was also highly effective in distinguishing NPS cultivars from other fruit types (Fig. 6). Notably, the consistency rate of 97.35% for the 21QP381 marker and 96.7% for the 23QP715 marker in distinguishing NPS fruit provides confidence in their applicability for efficient selection of desirable fruit color traits in eggplant (Fig. 6). Furthermore, combining these markers with our previously published markers like 22QP296 [28], which is linked to chlorophyll content in the fruit peel (Fig. 6), further enhances the potential for MAS in improving both pigmentation and overall fruit quality.

## Conclusion

In conclusion, this study identifies *SmMYB113* as a key regulator of light-independent anthocyanin accumulation in NPS eggplant, providing a valuable target for marker-assisted breeding of fruit color traits. The variation in RU repeats in the promoter region of *SmMYB113* in NPS eggplants likely upregulates its expression, leading to increased anthocyanin accumulation in a light-independent manner. In addition, the high-density genetic map and linked markers developed in this research offer a powerful toolkit for future eggplant breeding programs aimed at improving fruit color and quality. The findings also contribute to our understanding of the molecular basis of pigmentation in eggplant, offering new insights into the regulatory mechanisms that control anthocyanin biosynthesis in a light-independent manner.

## Materials and methods

### Plant materials, growth conditions, and sampling

The high-generation inbred lines of “””*S. melongena* used in this study were developed through conventional breeding methods at South China Agricultural University. These lines included 21E26, 21E27, 22E81, 22E82, 22E85, 24AE005, and 24AE006. The phenotypes of these lines are as follows: 21E26, 22E81, and 24AE006 bear green fruit; 22E82 produces white fruit; 22E85 produces photosensitive purple fruit; and 21E27 and 24AE005 bear NPS purple-black fruit. The 264 natural eggplant varieties were obtained from Guangzhou Jiaoyang Agriculture Co., Ltd. farm, Guangzhou. The 264 varieties were all planted in autumn 2021. The maturity fruits were used for phenotype analysis.

The populations for gene mapping were derived from a cross between 21E26 and 21E27. The 21E30 F_2_ population (21E26 × 21E27) was grown in the fall of 2020 at South China Agricultural University, Guangzhou. The derived F_3_, F_4_, and F_5_ populations, as well as the 24AE009 F_2_ population (24AE005 × 24AE006), were grown in the spring of 2024 at Guangzhou Jiaoyang Agriculture Co., Ltd. farm, Guangzhou.

For eggplant, after pollination, flowers were immediately covered with light-impermeable paper bags to exclude light for 14 days to facilitate fruit development in complete darkness. Subsequently, the bottom half of the fruit bags were removed, and after seven more days, fruits were sampled for color analysis, anthocyanin quantification, and gene expression studies. For tomato, flowers were bagged in a similar manner but for 25 days. Fruit peels from both species were collected, snap-frozen in liquid nitrogen, and stored at −80°C until further analysis.

### Extraction and quantification of chlorophyll and anthocyanins

To measure the contents of chlorophyll and anthocyanins, fruit peels were collected from at least three individual plants per biological replicate. Three biological replicates were used in the analyses. The extraction and quantification protocols for chlorophyll and anthocyanins followed previously described methods from our lab [9, 28].

### DNA isolation, bulk DNA library construction, and QTL-seq analysis

Genomic DNA was extracted from young true leaves of plants using the CTAB method. The DNA was then used to construct bulk DNA libraries. To construct the ‘NPS’ and ‘Green’ pools for QTL-sequencing, 20 plants exhibiting predominantly purple-black NPS fruit and 20 plants with green fruit were selected from the 21E30 F_2_ population.

Next, 150 bp pair-end sequencing libraries with insert ~500 bp fragment were prepared for resequencing on the Illimina HiSeq 2000 platform (BerryGenomics, Beijing, China). The QTL-seq analysis was performed using the QTL-seq program with the following parameters [37]: qtlseq -r HQ1315-reference.fasta -p 21E27.R1.fastq.gz,21E27.R2.fastq.gz -b1 NPS-pool.R1.fastq.gz,NPS-pool.R2.fastq.gz -b2 Green-pool.R1.fastq.gz,Green-pool.R2.fastq.gz -n1 20 -n2 20 -o NPS_dir -t 30 -T. The HQ1315 genome version was used as the reference for QTL-seq analysis [3].

### KASP marker development and genotyping

Parental lines 21E26 and 21E27 were re-sequenced as described in the QTL-seq section. After removing adaptors and low-quality reads, clean reads were processed to call SNPs using our lab’s custom script. High-quality SNPs identified between the two parental lines were used to develop fluorescence-based KASP markers using the SNP Primer design tool (http://www.snpway.com/). According to the published study [38], the optimum Tm of the two allele-specific primers was 60°C; the desired PCR product size was 80–200 bp; each primer should better have less than five repeating nucleotides in a row. The KASP markers used in this study are summarized in Table S3.

KASP genotyping was performed in a 384-well plate format with a total reaction volume of 5 μL, containing 2.5 μL of 2× KASP PCR mix (Gentides, Cat. No. E001-4), 0.075 μL of forward primer, 0.2 μL of reverse primer, 2 μL of genomic DNA (20–100 ng/μL), and ddH_2_O. The PCR conditions were 94°C for 15 min, followed by 10 cycles of 94°C for 20 s, 78°C for 10 s, and 65°C for 1 min, then 30 cycles of 94°C for 20 s and 57°C for 1 min. Fluorescent end-point readings were obtained using the Bio-Rad CFX384® Real-Time System (BioRad, Hercules, CA, USA).

### Linkage map construction and QTL mapping

Genetic distances between markers were estimated using the MAP function in QTL Ici-Mapping 4.2 software. The Kosambi map function was used to calculate map distances in centimorgans (cM). For QTL analysis, Bayesian Interval Mapping (BIP) within QTL Ici-Mapping 4.2 was employed, with a logarithm of odds (LOD) threshold value of 2.5 to define significant QTLs.

### Total RNA isolation, cDNA synthesis, and quantitative reverse transcription PCR

Total RNA was extracted from fruit peels using the Eastep® Super Kit (Cat. No. LS1040, Promega). cDNA was synthesized using the GoScript™ Reverse Transcription System Kit (Cat. No. A5001, Promega). Quantitative reverse transcription PCR (qRT-PCR) was conducted as previously described (Yan *et al.* [9]), using *SmCyclophilin* as the reference gene. Three technical replicates were performed for each sample, and gene expression was calculated using the 2^−ΔCt^ method. For gene expression analyses, fruit peels were collected from at least three individual plants to represent a biological replicate, with three biological replicates for each condition. Primers used for qRT-PCR are listed in Table S3.

### Cis-acting element analysis

Cis-acting elements in the RU region of the *SmMYB113* promoter were analyzed using the PlantCare database (http://bioinformatics.psb.ugent.be/webtools/plantcare/html/) to identify potential regulatory elements. Visualizations of read alignments around *SmMYB113* were done using the IGV [39]. The HQ1315 [3], 67/3 [40], and guiqie1 [41] genome was used as the reference for analysis, respectively.

### Plasmid construction and plant transformation

Genomic fragments of *SmMYB113* (including the promoter and coding region) from 21E26, 21E27, and 22E85 were amplified using KOD FX polymerase (Cat. No. TY-KFX-101, TOYOBO). The purified PCR products were then inserted into the modified binary vector pCambia2300, which lacks the 35S promoter. The resulting plasmids were introduced into the Micro-Tom cultivar and 21E26 by Agrobacterium tumefaciens (GV3101)-mediated transformation, respectively, as previously described [19, 27]. Transgenic plants were confirmed by PCR using NTP II-specific primers. The primers used for plasmid construction and transgenic plant confirmation are listed in Table S3.

### Statistical analyses

Statistical analyses were conducted using IBM SPSS Statistics 23. One-way Analysis of Variance (ANOVA) and *t*-tests were performed in Excel to assess the significance of differences between experimental groups.

## Supplementary Material

Web_Material_uhaf319

## Data Availability

The data that support the findings of this study are available in the Supporting Information.
