# Supplementary material for: A 725-bp quadruple repeat in the promoter of SmMYB113 is associated with light-independent anthocyanin regulation in eggplant
Source: Hortic Res. 2025 Nov 21;13(3):uhaf319. doi: 10.1093/hr/uhaf319 (PMC12962852; doi:10.1093/hr/uhaf319)
Supplement: Web_Material_uhaf319 [file web_material_uhaf319.zip › Figure S2.pdf]

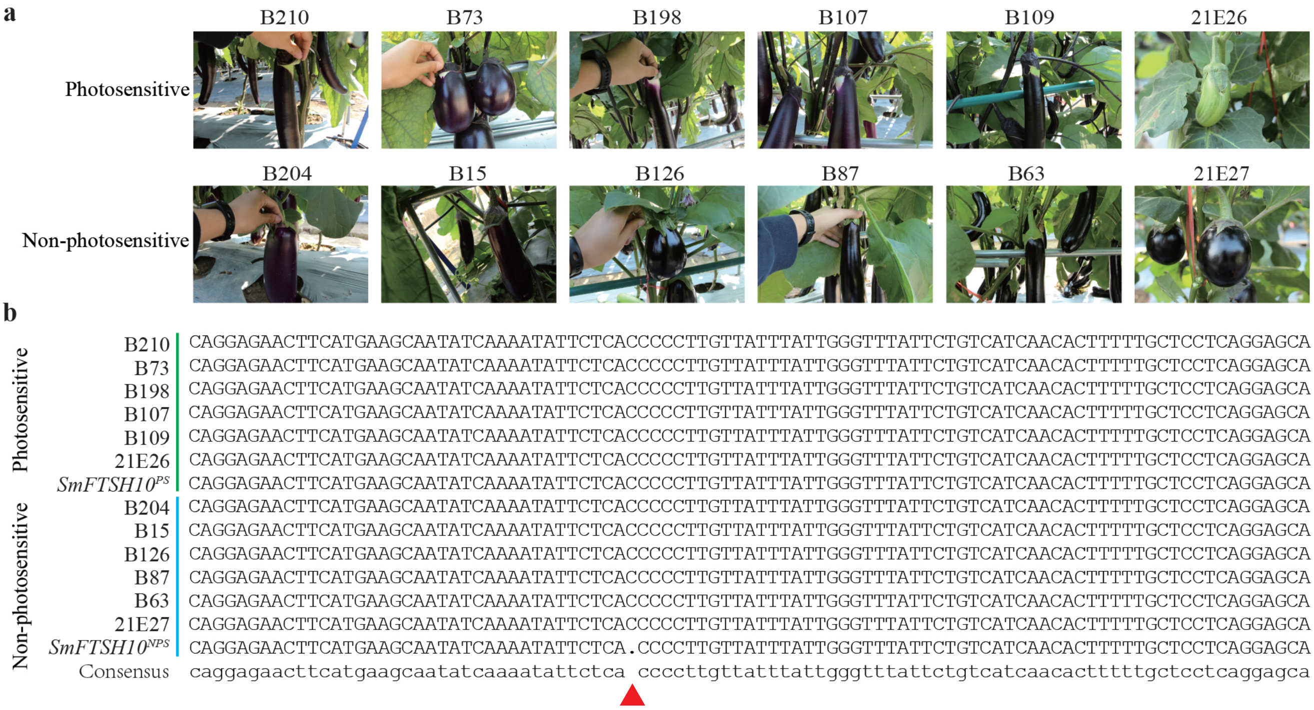
a
Photosensitive
B210
B73
B198
B107
B109
21E26
Non-photosensitive
B204
B15
B126
B87
B63
21E27
b
Photosensitive
Non-photosensitive
B210 CAGGAGAACTTCATGAAGCAATATCAAAATATTCTCACCCCCTTGTTATTTATTGGGTTTATTCTGTCATCAACACTTTTTGCTCCTCAGGAGCA
B73 CAGGAGAACTTCATGAAGCAATATCAAAATATTCTCACCCCCTTGTTATTTATTGGGTTTATTCTGTCATCAACACTTTTTGCTCCTCAGGAGCA
B198 CAGGAGAACTTCATGAAGCAATATCAAAATATTCTCACCCCCTTGTTATTTATTGGGTTTATTCTGTCATCAACACTTTTTGCTCCTCAGGAGCA
B107 CAGGAGAACTTCATGAAGCAATATCAAAATATTCTCACCCCCTTGTTATTTATTGGGTTTATTCTGTCATCAACACTTTTTGCTCCTCAGGAGCA
B109 CAGGAGAACTTCATGAAGCAATATCAAAATATTCTCACCCCCTTGTTATTTATTGGGTTTATTCTGTCATCAACACTTTTTGCTCCTCAGGAGCA
21E26 CAGGAGAACTTCATGAAGCAATATCAAAATATTCTCACCCCCTTGTTATTTATTGGGTTTATTCTGTCATCAACACTTTTTGCTCCTCAGGAGCA
SmFTSH10PS CAGGAGAACTTCATGAAGCAATATCAAAATATTCTCACCCCCTTGTTATTTATTGGGTTTATTCTGTCATCAACACTTTTTGCTCCTCAGGAGCA
B204 CAGGAGAACTTCATGAAGCAATATCAAAATATTCTCACCCCCTTGTTATTTATTGGGTTTATTCTGTCATCAACACTTTTTGCTCCTCAGGAGCA
B15 CAGGAGAACTTCATGAAGCAATATCAAAATATTCTCACCCCCTTGTTATTTATTGGGTTTATTCTGTCATCAACACTTTTTGCTCCTCAGGAGCA
B126 CAGGAGAACTTCATGAAGCAATATCAAAATATTCTCACCCCCTTGTTATTTATTGGGTTTATTCTGTCATCAACACTTTTTGCTCCTCAGGAGCA
B87 CAGGAGAACTTCATGAAGCAATATCAAAATATTCTCACCCCCTTGTTATTTATTGGGTTTATTCTGTCATCAACACTTTTTGCTCCTCAGGAGCA
B63 CAGGAGAACTTCATGAAGCAATATCAAAATATTCTCACCCCCTTGTTATTTATTGGGTTTATTCTGTCATCAACACTTTTTGCTCCTCAGGAGCA
21E27 CAGGAGAACTTCATGAAGCAATATCAAAATATTCTCACCCCCTTGTTATTTATTGGGTTTATTCTGTCATCAACACTTTTTGCTCCTCAGGAGCA
SmFTSH10NPS CAGGAGAACTTCATGAAGCAATATCAAAATATTCTCA.CCCCTTGTTATTTATTGGGTTTATTCTGTCATCAACACTTTTTGCTCCTCAGGAGCA
Consensus caggagaacttcatgaagcaatatcaaaatattctca ccccttgttatttattgggtttattctgtcatcaacactttttgctcctcaggagca
