# Supplementary material for: A 725-bp quadruple repeat in the promoter of SmMYB113 is associated with light-independent anthocyanin regulation in eggplant
Source: Hortic Res. 2025 Nov 21;13(3):uhaf319. doi: 10.1093/hr/uhaf319 (PMC12962852; doi:10.1093/hr/uhaf319)
Supplement: Web_Material_uhaf319 [file web_material_uhaf319.zip › Figure S8.pdf]

```
21E27  ATGAATAATCCTCCTATAATCTGTACGTCTGTGCGAGTGAGGAAAGGTTCATGGACTGA
21E26  ATGAATAATCCTCCTATAATCTGTACGTCTGTGCGAGTGAGGAAAGGTTCATGGACTGA
22E85  ATGAATAATCCTCCTATAATCTGTACGTCTGTGCGAGTGAGGAAAGGTTCATGGACTGA
guiqie1 ATGAATAATCCTCCTATAATCTGTACGTCTGTGCGAGTGAGGAAAGGTTCATGGACTGA
HQ1315 ATGAATAATCCTCCTATAATCTGTACGTCTGTGCGAGTGAGGAAAGGTTCATGGACTGA

21E27  AGAAGAAGATTTACTCTTGAGGAAATGTATGGAAAAATATGGTGAAGGCAAGTGGCATC
21E26  AGAAGAAGATTTACTCTTGAGGAAATGTATGGAAAAATATGGTGAAGGCAAGTGGCATC
22E85  AGAAGAAGATTTACTCTTGAGGAAATGTATGGAAAAATATGGTGAAGGCAAGTGGCATC
guiqie1 AGAAGAAGATTTACTCTTGAGGAAATGTATGGAAAAATATGGTGAAGGCAAGTGGCATC
HQ1315 AGAAGAAGATTTACTCTTGAGGAAATGTATGGAAAAATATGGTGAAGGCAAGTGGCATC

21E27  TTGTTCCTGCTAGAGCAGGTCTGAATAGATGCAGAAAAAGTTGTAGGCTGAGGTGGTTG
21E26  TTGTTCCTGCTAGAGCAG-----------------------------------------
22E85  TTGTTCCTGCTAGAGCAGGTCTGAATAGATGCAGAAAAAGTTGTAGGCTGAGGTGGTTG
guiqie1 TTGTTCCTGCTAGAGCAGGTCTGAATAGATGCAGAAAAAGTTGTAGGCTGAGGTGGTTG
HQ1315 TTGTTCCTGCTAGAGCAGGTCTGAATAGATGCAGAAAAAGTTGTAGGCTGAGGTGGTTG

21E27  AATTATTTAAGGCCACATATCAAGAGAGGTGACTTTGCTTCGGATGAAGTGGATCTCAT
21E26  -----------------------------------------------------------
22E85  AATTATTTAAGGCCACATATCAAGAGAGGTGACTTTGCTTCGGATGAAGTGGATCTCAT
guiqie1 AATTATTTAAGGCCACATATCAAGAGAGGTGACTTTGCTTCGGATGAAGTGGATCTCAT
HQ1315 AATTATTTAAGGCCACATATCAAGAGAGGTGACTTTGCTTCGGATGAAGTGGATCTCAT

21E27  CTTGAGGCTTCATAAGCTCTTAGGCAACAGATGGTCACTTATTGCTGGTAGACTTCCGG
21E26  ------------------------------ATGGTCACTTATTGCTGGTAGACTTCCGG
22E85  CTTGAGGCTTCATAAGCTCTTAGGCAACAGATGGTCACTTATTGCTGGTAGACTTCCGG
guiqie1 CTTGAGGCTTCATAAGCTCTTAGGCAACAGATGGTCACTTATTGCTGGTAGACTTCCGG
HQ1315 CTTGAGGCTTCATAAGCTCTTAGGCAACAGATGGTCACTTATTGCTGGTAGACTTCCGG

21E27  GAAGGACCGCAAACGATGTAAAGAACTACTGGAACACTAACCTTCTAAGGAAGTTCACT
21E26  GAAGGACCGCAAACGATGTAAAGAACTACTGGAACACTAACCTTCTAAGGAAGTTCACT
22E85  GAAGGACCGCAAACGATGTAAAGAACTACTGGAACACTAACCTTCTAAGGAAGTTCACT
guiqie1 GAAGGACCGCAAACGATGTAAAGAACTACTGGAACACTAACCTTCTAAGGAAGTTCACT
HQ1315 GAAGGACCGCAAACGATGTAAAGAACTACTGGAACACTAACCTTCTAAGGAAGTTCACT

21E27  ATTGCTCCTCAAAAGATTAATAATACATGCAAAGACATCATTAGTACGAATGAAATAAT
21E26  ATTGCTCCTCAAAAGATTAATAATACATGCAAAGACATCATTAGTACGAATGAAATAAT
22E85  ATTGCTCCTCAAAAGATTAATAATACATGCAAAGACATCATTAGTACGAATGAAATAAT
guiqie1 ATTGCTCCTCAAAAGATTAATAATACATGCAAAGACATCATTAGTACGAATGAAATAAT
HQ1315 ATTGCTCCTCAAAAGATTAATAATACATGCAAAGACATCATTAGTACGAATGAAATAAT

21E27  AAGACCTCAACCTCGGAAA-TACTTGTCAAGCATAAAGAAGAATAATTTGACAAACAAT
21E26  AAGACCTCAACCTCGGAAAATACTTGTCAAGCATAAAGAAGAATAATTTGACAAACAAT
22E85  AAGACCTCAACCTCGGAAA-TACTTGTCAAGCATAAAGAAGAATAATTTGACAAACAAT
guiqie1 AAGACCTCAACCTCGGAAA-TACTTGTCAAGCATAAAGAAGAATAATTTGACAAACAAT
HQ1315 AAGACCTCAACCTCGGAAA-TACTTGTCAAGCATAAAGAAGAATAATTTGACAAACAAT

21E27  AATGTAATTGTAGACAAGGAAGAACGCTGCAAAGAAATAACAAGTGACAAGCAAACTAC
21E26  AATGTAATTGTAGACAAGGAAGAACGCTGCAAAGAAATAACAAGTGACAAGCAAACTAC
22E85  AATGTAATTGTAGACAAGGAAGAACGCTGCAAAGAAATAACAAGTGACAAGCAAACTAC
guiqie1 AATGTAATTGTAGACAAGGAAGAACGCTGCAAAGAAATAACAAGTGACAAGCAAACTAC
HQ1315 AATGTAATTGTAGACAAGGAAGAACGCTGCAAAGAAATAACAAGTGACAAGCAAACTAC

21E27  CGATGCATCGATGGATAACGGAGATCAATGGTGGAAAAGTTTACTGGAAAATTTCAATG
21E26  CGATGCATCGATGGATAACGGAGATCAATGGTGGAAAAGTTTACTGGAAAATTTCAATG
22E85  CGATGCATCGATGGATAACGGAGATCAATGGTGGAAAAGTTTACTGGAAAATTTCAATG
guiqie1 CGATGCATCGATGGATAACGGAGATCAATGGTGGAAAAGTTTACTGGAAAATTTCAATG
HQ1315 CGATGCATCGATGGATAACGGAGATCAATGGTGGAAAAGTTTACTGGAAAATTTCAATG

21E27  ACGACGCTGTTGAAGGAGAAGAAGAAGCTGTAACTAATTATGAAAAAACACTAACAAGT
21E26  ACGACGCTGTTGAAGGAGAAGAAGAAGCTGTAACTAATTATGAAAAAACACTAACAAGT
22E85  ACGACGCTGTTGAAGGAGAAGAAGAAGCTGTAACTAATTATGAAAAAACACTAACAAGT
guiqie1 ACGACGCTGTTGAAGGAGAAGAAGAAGCTGTAACTAATTATGAAAAAACACTAACAAGT
HQ1315 ACGACGCTGTTGAAGGAGAAGAAGAAGCTGTAACTAATTATGAAAAAACACTAACAAGT

21E27  TTATTACATGAGGAAATATCATCACCACCATTAAATGGTGGAGGCAACTCCATGCAACA
21E26  TTATTACATGAGGAAATATCATCACCACCATTAAATGGTGGAGGCAACTCCATGCAACA
22E85  TTATTACATGAGGAAATATCATCACCACCATTAAATGGTGGAGGCAACTCCATGCAACA
guiqie1 TTATTACATGAGGAAATATCATCACCACCATTAAATGGTGGAGGCAACTCCATGCAACA
HQ1315 TTATTACATGAGGAAATATCATCACCACCATTAAATGGTGGAGGCAACTCCATGCAACA

21E27  AGAACAATGTGATAATTGGGATGATTTTTCTGCTGATATTGATTTATGGAATCTACTTG
21E26  AGAACAATGTGATAATTGGGATGATTTTTCTGCTGATATTGATTTATGGAATCTACTTG
22E85  AGAACAATGTGATAATTGGGATGATTTTTCTGCTGATATTGATTTATGGAATCTACTTG
guiqie1 AGAACAATGTGATAATTGGGATGATTTTTCTGCTGATATTGATTTATGGAATCTACTTG
HQ1315 AGAACAATGTGATAATTGGGATGATTTTTCTGCTGATATTGATTTATGGAATCTACTTG

21E27  ATTAA
21E26  ATTAA
22E85  ATTAA
guiqie1 ATTAA
HQ1315 ATTAA
```
