# Supplementary material for: A 725-bp quadruple repeat in the promoter of SmMYB113 is associated with light-independent anthocyanin regulation in eggplant
Source: Hortic Res. 2025 Nov 21;13(3):uhaf319. doi: 10.1093/hr/uhaf319 (PMC12962852; doi:10.1093/hr/uhaf319)
Supplement: Web_Material_uhaf319 [file web_material_uhaf319.zip › Figure S9.pdf]

```
guiqie1 MNNPPIICTSVRVRKGSWTEEEDLLLRKCMEKYGEGKWHLVPARAGLNRCRKSCRLRWL
  21E27 MNNPPIICTSVRVRKGSWTEEEDLLLRKCMEKYGEGKWHLVPARAGLNRCRKSCRLRWL
  21E26 MNNPPIICTSVRVRKGSWTEEEDLLLRKCMEKYGEGKWHLVPARA--------------
 HQ1315 MNNPPIICTSVRVRKGSWTEEEDLLLRKCMEKYGEGKWHLVPARAGLNRCRKSCRLRWL
  22E85 MNNPPIICTSVRVRKGSWTEEEDLLLRKCMEKYGEGKWHLVPARAGLNRCRKSCRLRWL

guiqie1 NYLRPHIKRGDFASDEVDLILRLHKLLGNRWSLIAGRLPGRTANDVKNYWNTNLLRKFT
  21E27 NYLRPHIKRGDFASDEVDLILRLHKLLGNRWSLIAGRLPGRTANDVKNYWNTNLLRKFT
  21E26 ----------------------------------DGHL---------------LLVDFR
 HQ1315 NYLRPHIKRGDFASDEVDLILRLHKLLGNRWSLIAGRLPGRTANDVKNYWNTNLLRKFT
  22E85 NYLRPHIKRGDFASDEVDLILRLHKLLGNRWSLIAGRLPGRTANDVKNYWNTNLLRKFT

guiqie1 IAPQKINNTCKDIISTNEIIRPQPRKYLSSIKKNNLTNNNVIVDKEERCKEITSDKQTT
  21E27 IAPQKINNTCKDIISTNEIIRPQPRKYLSSIKKNNLTNNNVIVDKEERCKEITSDKQTT
  21E26 EGPQTM-----------------------------------------------------
 HQ1315 IAPQKINNTCKDIISTNEIIRPQPRKYLSSIKKNNLTNNNVIVDKEERCKEITSDKQTT
  22E85 IAPQKINNTCKDIISTNEIIRPQPRKYLSSIKKNNLTNNNVIVDKEERCKEITSDKQTT

guiqie1 DASMDNGDQWWKSLLENFNDDAVEGEEEAVTNYEKTLTSLLHEEISSPPLNGGGNSMQQ
  21E27 DASMDNGDQWWKSLLENFNDDAVEGEEEAVTNYEKTLTSLLHEEISSPPLNGGGNSMQQ
  21E26 -----------------------------------------------------------
 HQ1315 DASMDNGDQWWKSLLENFNDDAVEGEEEAVTNYEKTLTSLLHEEISSPPLNGGGNSMQQ
  22E85 DASMDNGDQWWKSLLENFNDDAVEGEEEAVTNYEKTLTSLLHEEISSPPLNGGGNSMQQ

guiqie1 EQCDNWDDFSADIDLWNLLD
  21E27 EQCDNWDDFSADIDLWNLLD
  21E26 --------------------
 HQ1315 EQCDNWDDFSADIDLWNLLD
  22E85 EQCDNWDDFSADIDLWNLLD
```
