# Supplementary material for: A 725-bp quadruple repeat in the promoter of SmMYB113 is associated with light-independent anthocyanin regulation in eggplant
Source: Hortic Res. 2025 Nov 21;13(3):uhaf319. doi: 10.1093/hr/uhaf319 (PMC12962852; doi:10.1093/hr/uhaf319)
Supplement: Web_Material_uhaf319 [file web_material_uhaf319.zip › Supplementary Information-20250913-V2.docx]

**A 725 bp quadruple repeats unit in the promoter of *SmMYB113* is responsible for light-independent anthocyanin regulation in eggplant**

Zhilei Xia^1^, Meng Yang^1^, Yinggemei Huang^1^, Bingxin Yu^1^, Tingxia Wan^1^, Duanhua Wang^2^, Qian Li^2^, Manoj Sapkota^3^, Shuangshuang Yan^1^, Bihao Cao^1,*^, Zhengkun Qiu^1,*^

^1^College of Horticulture, Key Laboratory of Biology and Genetic Improvement of Horticultural Crops (South China), Ministry of Agriculture and Rural Affairs/Guangdong Vegetable Engineering and Technology Research Center, South China Agricultural University, Guangzhou, 510642, China

^2^Institute of Vegetables, Hunan Academy of Agricultural Sciences, Changsha, 416007, China

^3^Department of Horticulture, University of Kentucky, Lexington, KY, 40546, USA

*Corresponding authors: Zhengkun Qiu, Email: [qiuzhengkun@scau.edu.cn](mailto:qiuzhengkun@scau.edu.cn); Bihao Cao, Email: [caobh01@163.com](mailto:caobh01@163.com)

Zhilei Xia: 1749511906@qq.com

Meng Yang: ymmeng1018@163.com

Yinggemei Huang: 920892739@qq.com

Bingxin Yu: 13533120469@163.com

Tingxia Wan: [15915943752@163.com](mailto:15915943752@163.com)

Duanhua Wang: duanhuawang0559@163.com

Qian Li: 18390950946@139.com

Manoj Sapkota: Manoj.sapkota@uky.edu

Shuangshuang Yan: ssyan@scau.edu.cn

Bihao Cao: caobh01@163.com

Zhengkun Qiu: qiuzhengkun@scau.edu.cn

**Supplementary Information**


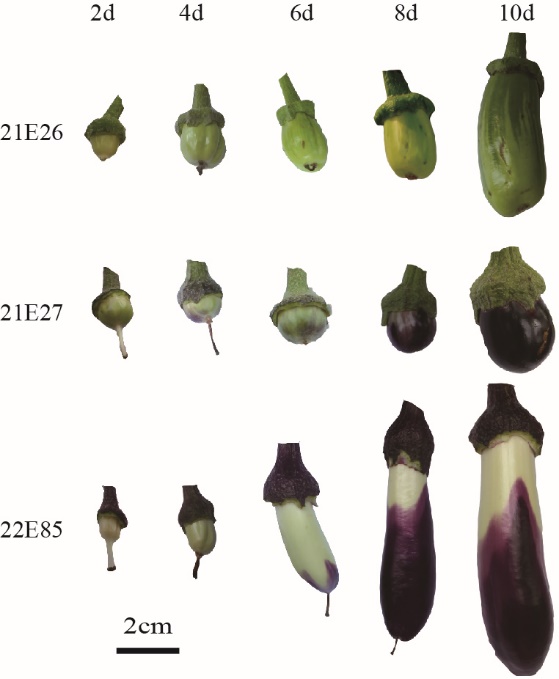


Fig. S1 Fruit phenotype of var. 21E26, 21E27 and 22E85 in different development stages. d means days after pollination. Sepals were removed to show the color under sepal of eggplant.


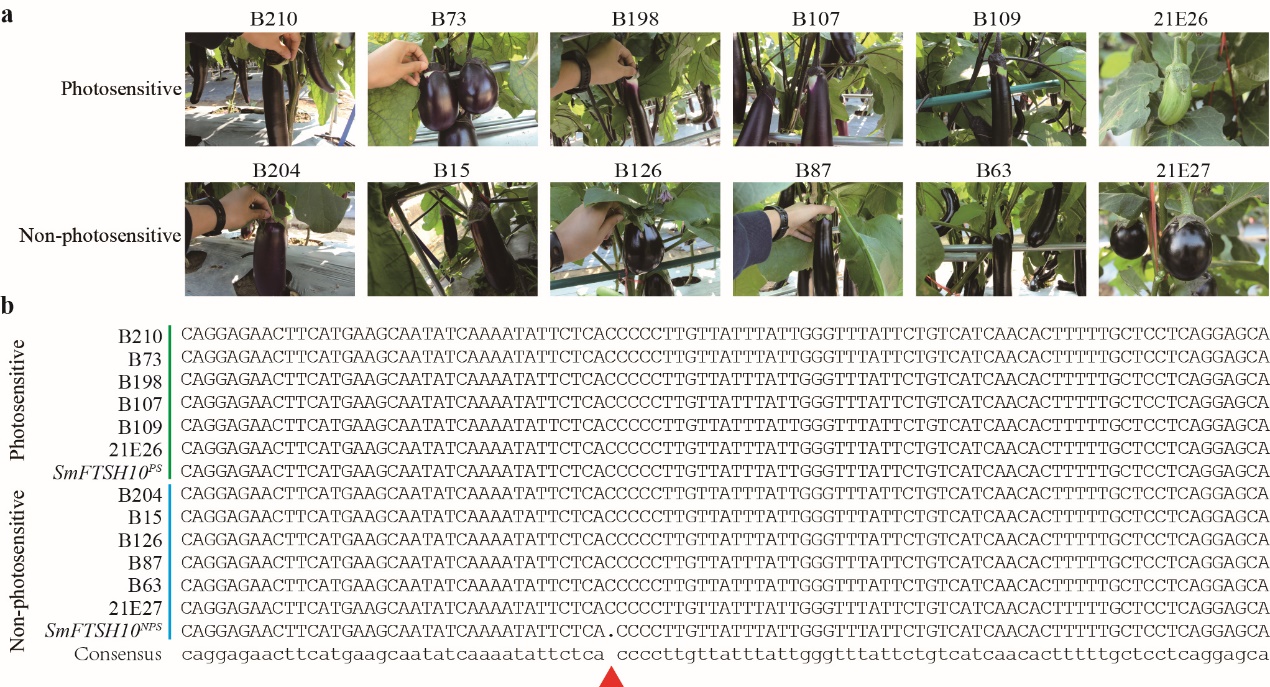


Fig. S2 Phenotype (a) and genotype of *SmFTSH10* (b) analysis of some photosensitive and non-photosensitive eggplant varieties. The red triangle shows the position of C-base deletion in *SmFTSH10* in non-photosensitive eggplant. *SmFTSH10^PS^* means the genotype of *SmFTSH10* in photosensitive eggplant, and *SmFTSH10^NPS^* means the genotype of *SmFTSH10* in non-photosensitive eggplant


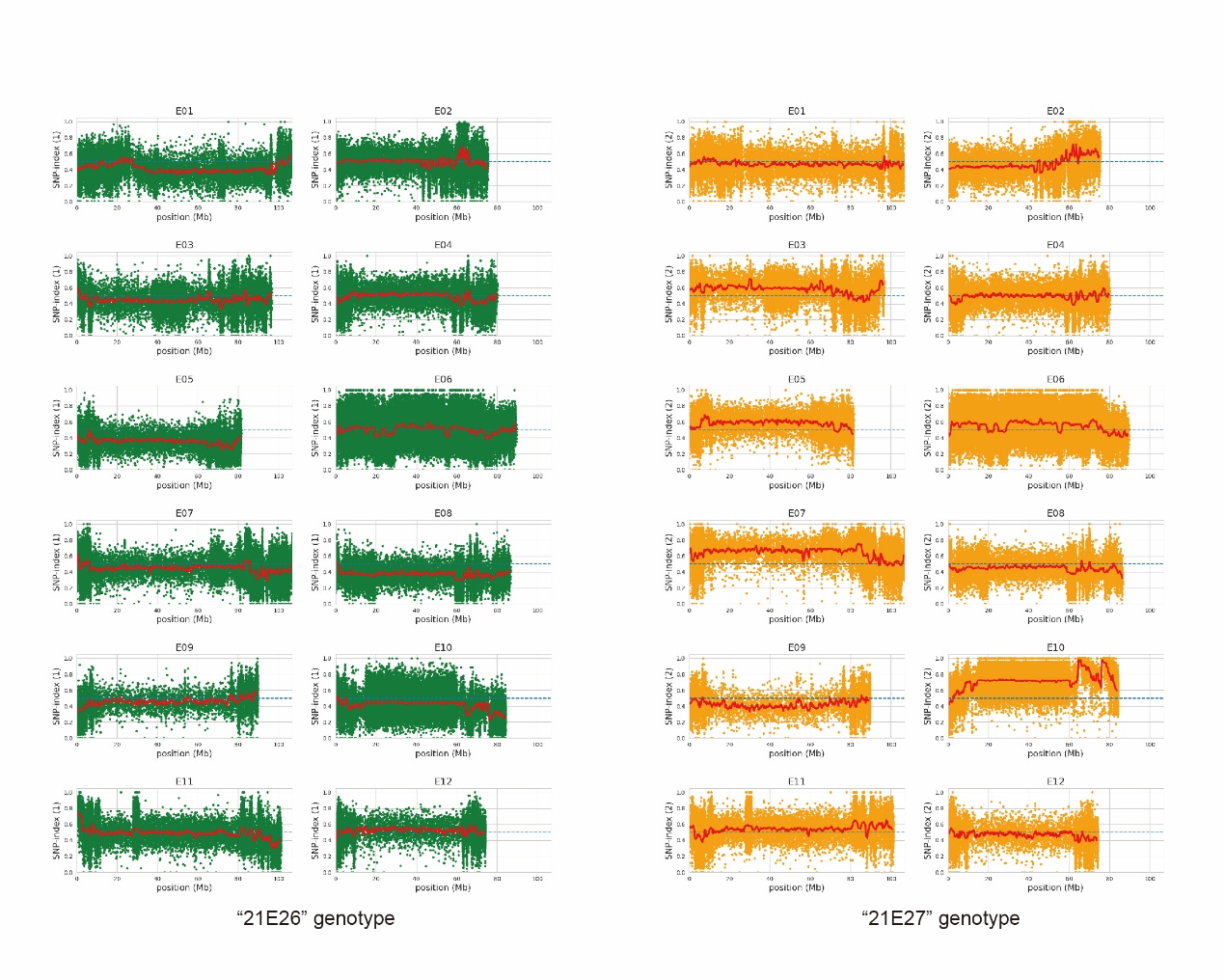


Fig. S3 SNP-index plots of the DNA bulks. NPS, non-photosensitive.


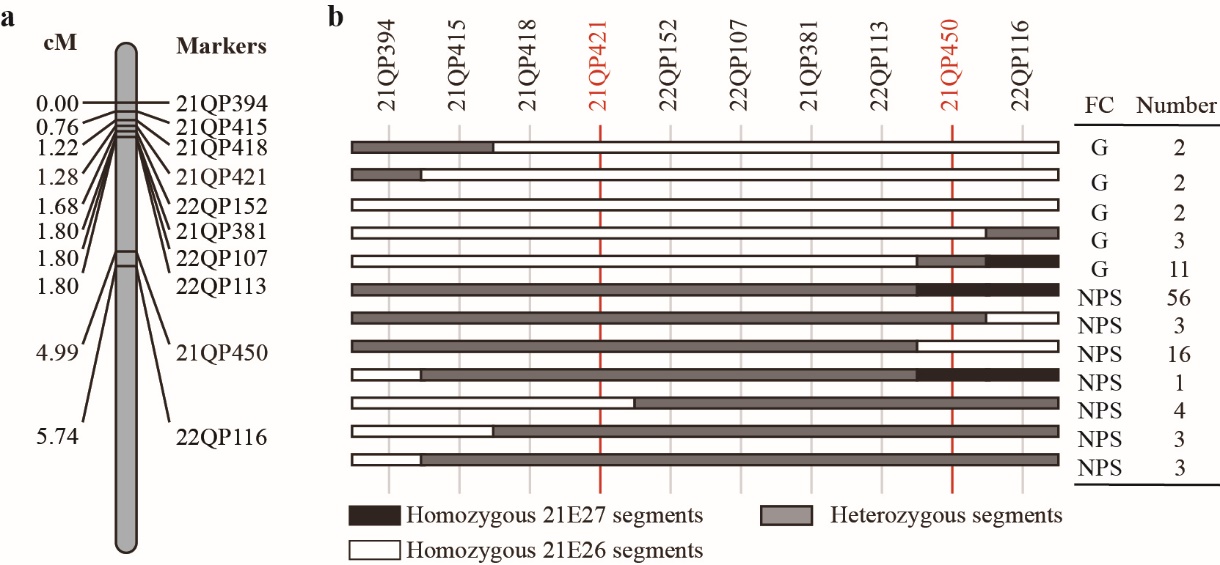


Fig. S4 Mapping of *SmNPS10.1* in the F_2:3_ population. a. Linkage map and map distances of the markers that used for *SmNPS10.1* mapping. b. The genotype and fruit color of the recombinants. FC means fruit color, G means green fruit, NPS means non-photosensitive fruit.


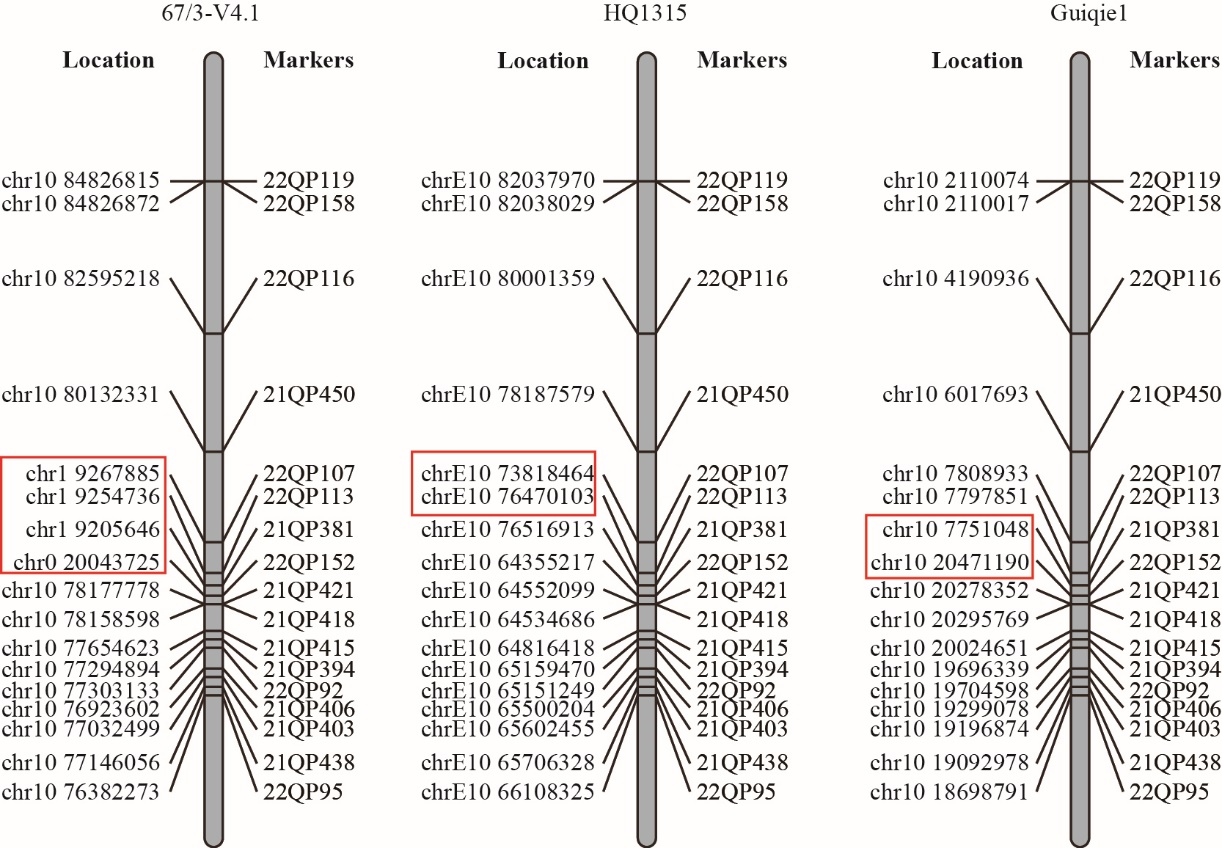


Fig. S5 The genomic positions of the markers in 67/3, HQ1315 and guiqie1 genome references. The red quadrate shows the *SmNPS10.1* mapping region.


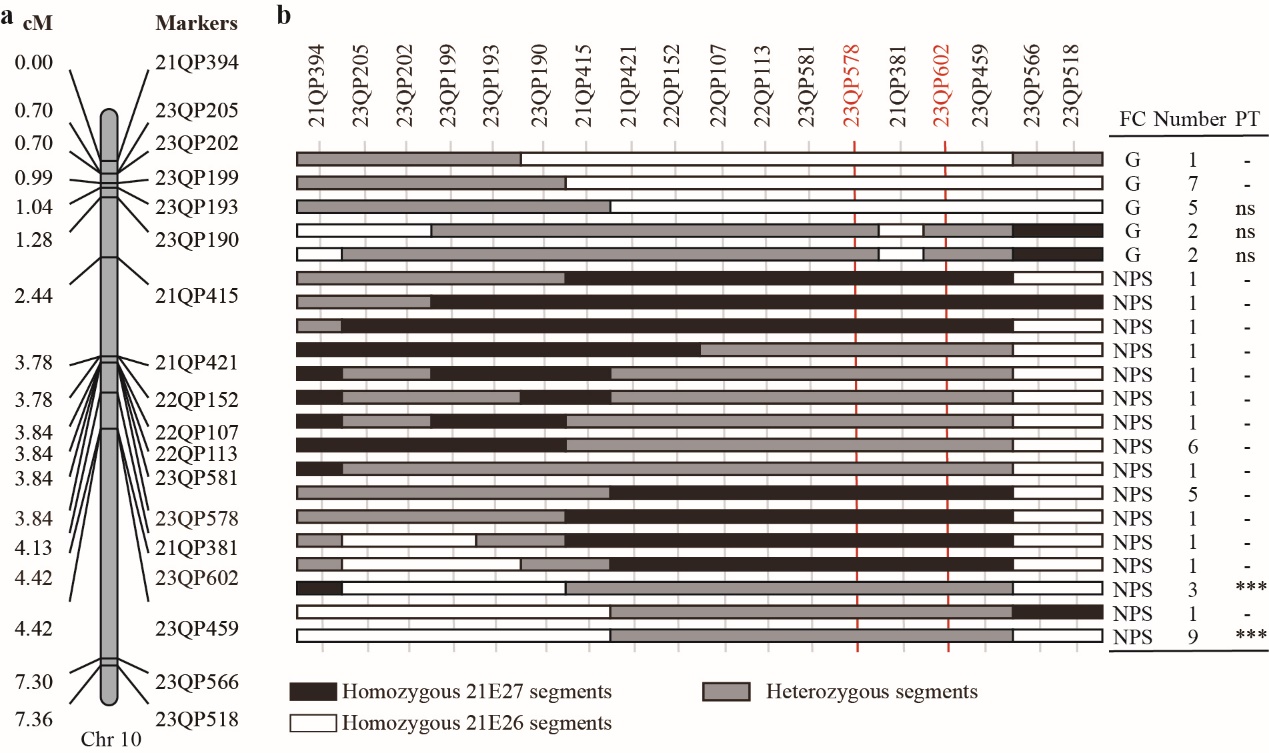


Fig. S6 Mapping of *SmNPS10.1* in the F_3:4_ population. a. Linkage map and map distances of the markers that used for *SmNPS10.1* mapping. b. The genotype and fruit color of the recombinants. FC means fruit color, G means green fruit, NPS means non-photosensitive fruit, PT means progeny test, ns means no significant, *** means P<0.001 (Student’s t-test), - means lines were not selected for PT.


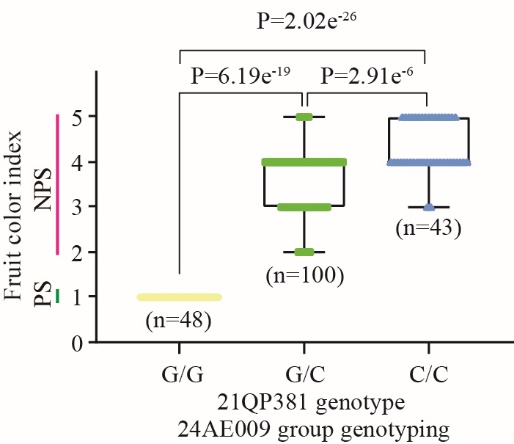
Fig. S7 Coincidence analysis between the phenotype and genotype of KASP marker 21QP381 in 24AE009 F_2_ population. PS means photosensitive fruit, NPS non-photosensitive fruit.


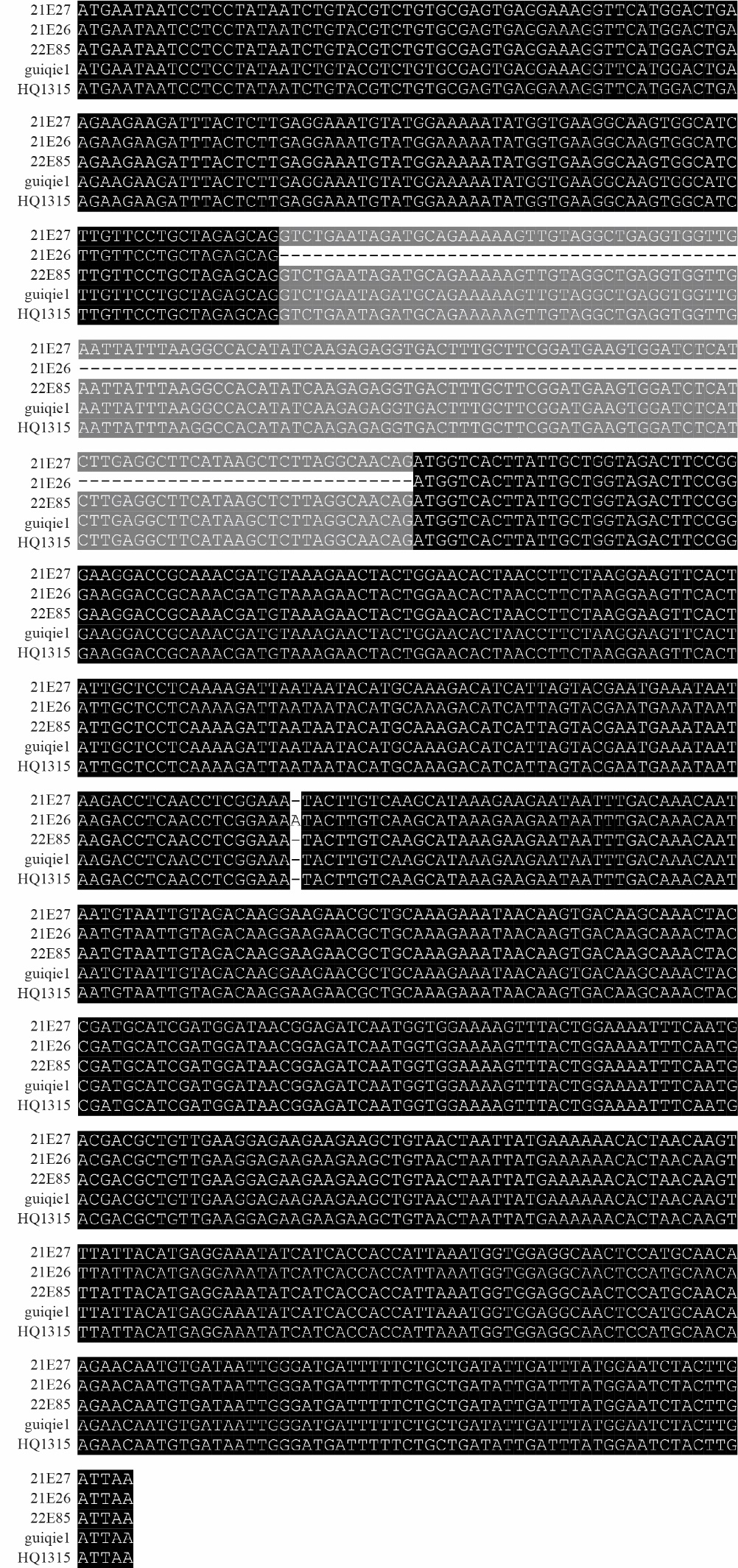


Fig. S8 Genomic sequence alignment of *SmMYB113* in var. 21E26, 21E27, 22E85, guiqie1 and HQ1315.


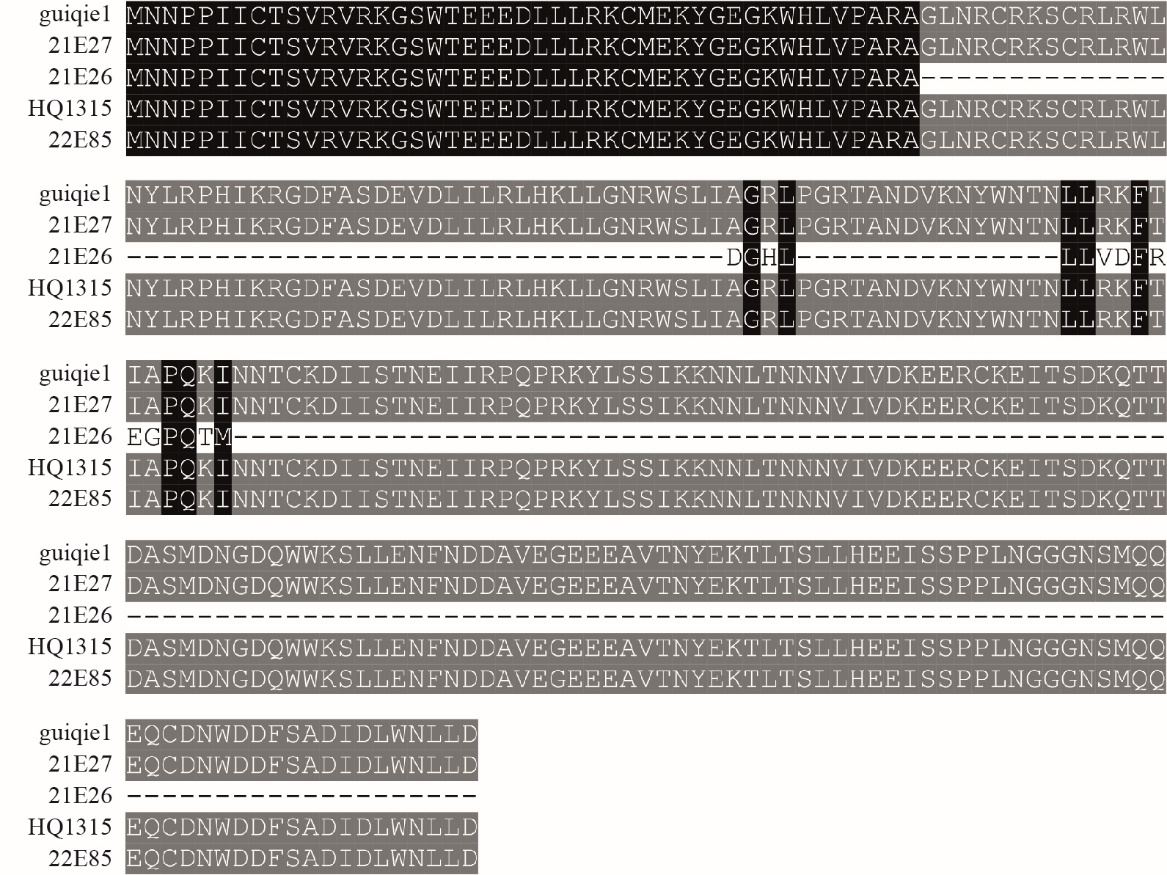


Fig. S9 Predicted protein sequence alignment of *SmMYB113* in var. 21E26, 21E27, 22E85, guiqie1 and HQ1315.


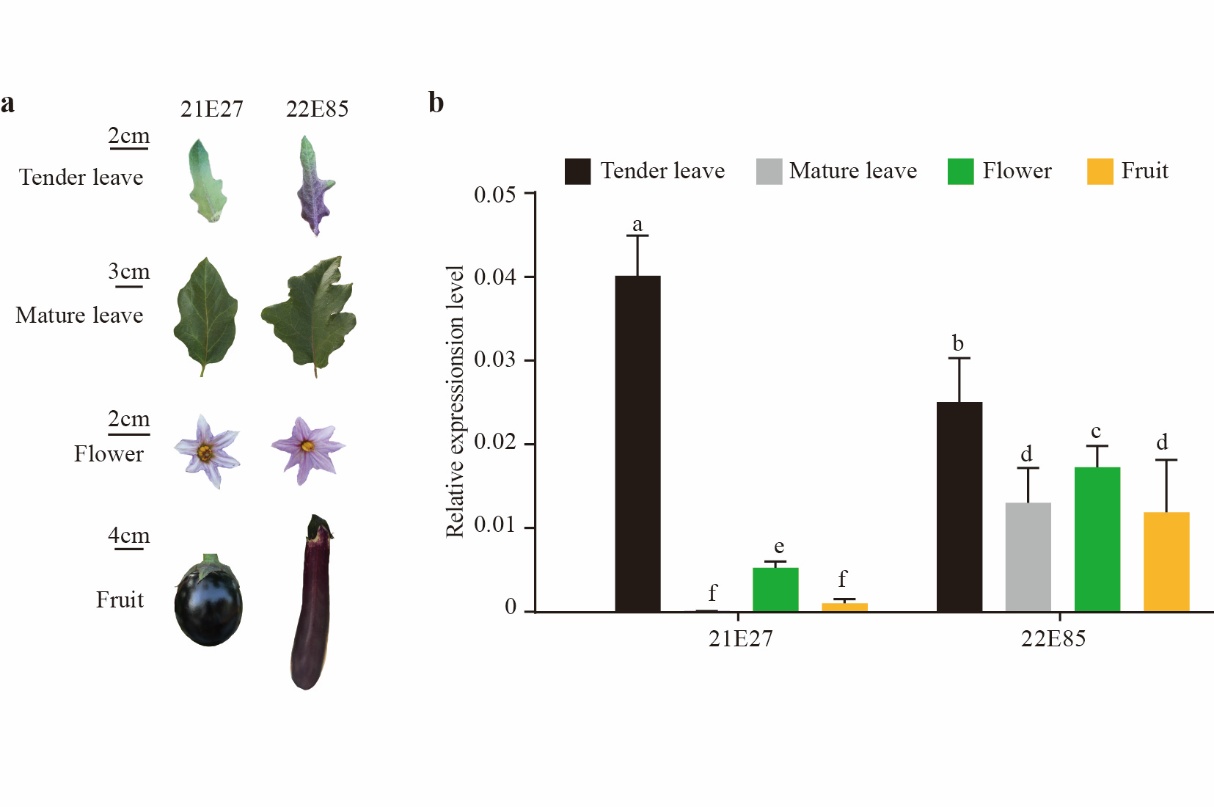


Fig. S10. Expression pattern analysis of SmMYB113 in tender leaves, mature leaves, flowers, and fruit peels of 21E27 and 22E85. a. Phenotypes of tender leaves, mature leaves, flowers, and fruit of 21E27 and 22E85 accessions. b. Expression patterns of SmMYB113 in tender leaves, mature leaves, flowers, and fruit peels of 21E27 and 22E85. Different letters indicate statistically signiﬁcant differences among groups (Tukey’s honest signiﬁcant difference test, P < 0.05).


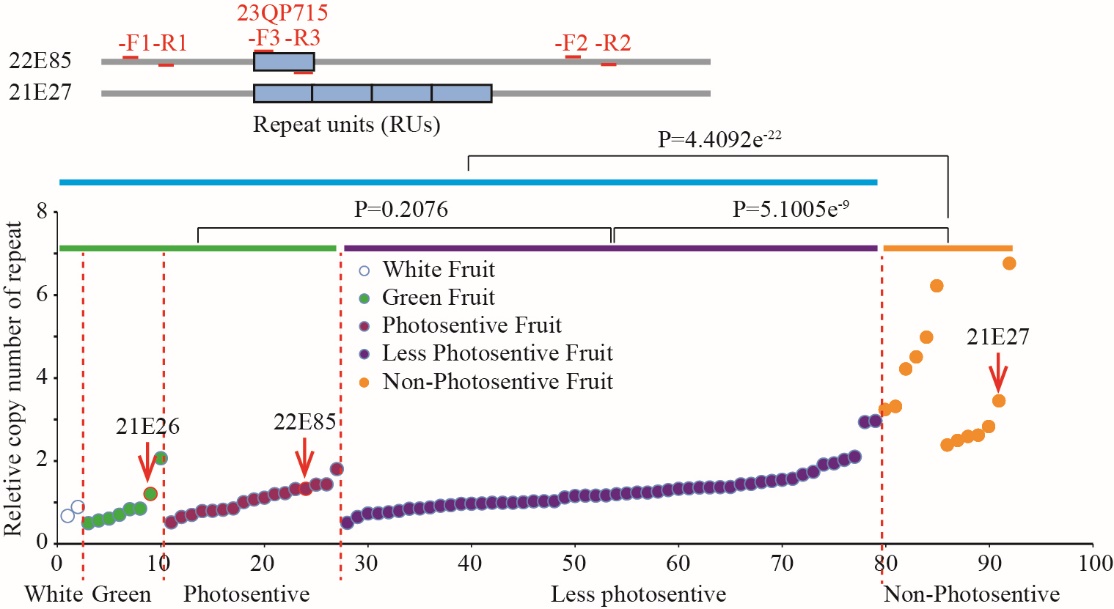


Fig. S11 Coincidence analysis between the phenotype and genotype of marker 23QP715 in natural eggplant varieties. The genotype of 23QP715 was shown in a relative copy number of the repeat units. Relative copy number of the repeat units=copy number of the 23QP715-F3-R3 PCR products/ average of copy number of the (23QP715-F1-R1 PCR products and 23QP715-F2-R2 PCR products).


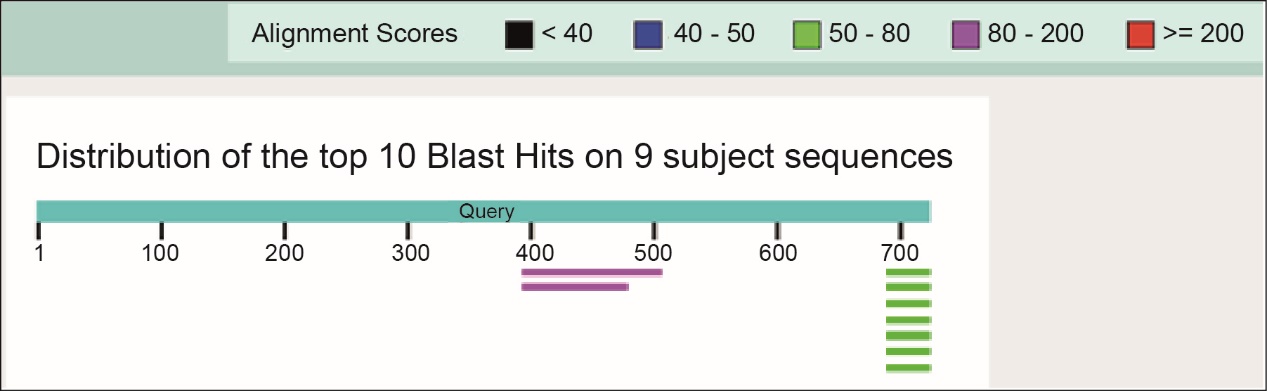


Fig. S12 Blast analysis with the sequence of repeat unit in National Center for Biotechnology Information (NCBI).

**Table S1. Sequence of repeat unit in promoter of *SmMYB113*.**

AGTAAAATATTATTTAATTATATTATTAAAAAATATTGACTTTTAGATCTGTGCATTGCACAAAATTATCGTAACTACCAAATTAATTAATGGTACTCTAGTTATGTCATATGCCAATCATTTTTTGACTTTTTTAATTTCCTCCATTATTAATATTATCCATGTGGTGGAAAGGGGGTCAAGAAACAATCCTATTTGGTCACTACCAAACTATTTTTTATTTTTACCAAATTAAATAATGGTACTTTAGTTGGGCCATATGTCACTTGTTTTTTGATCATTCGAGTAATTTGCAATTTGCACCCATAATATATGCGTTTTTTGTAATTTGCCCCTTATTTTATCATTTTTTGTGATTTGCACCTTAATTTTTTTATTTCCTTACAAAATTGAGTATAATTTGTGATTTGCACCCCTAATGTATGCCCTTTTTTGTGGCTTGCACCTTATTCTATCATTTTTTGTGATTGGCACCTCAATCTTTTTATTTCATTGCAAAATCATAAAAATTTAAAAAAAAAATCCAACAATTTTCAAAGGGTAAACAAGGAAAACAATGAATATTTCTATAAAAAGTATTTTATTCACGAGAAAATTAGGTGGATCCTCTTCTTCTATCTTGGCAACATATAAAACAATTTAAAGGAAGCTTAAGTTTTGATTGTAAAAATGAAACCCATATTTTTTTTGTGAGCCAAGAAAATGAAGCAGGAGAGGGAGATGAT

**Table S2. Cis-element analysis in the repeat unit region.**

| cis-acting elements | Sequences | Functions |
| --- | --- | --- |
| AAAC-motif | TTCAAAACTAAC | light responsive element |
| AE-box | AGAAACAA | part of a module for light response |
| AT1-motif | AATTATTTTTTATT | part of a light responsive module |
| Box 4 | ATTAAT | part of a conserved DNA module involved in light responsiveness |
| I-box | GATAAGGTG | part of a light responsive element |
| TCCC-motif | TCTCCCT | part of a light responsive element |

**Table S3. Primers used in this study.**

| **KASP primers used for gene mapping** | | | | |
| --- | --- | --- | --- | --- |
| Marker Name | Position | F/R | | Sequence（5′~3′） |
| 21QP381 | chrE10 76516913 | F_P1_ | | GAAGGTGACCAAGTTCATGCTCAAAACATCCAAATTTGCCCATGG |
|  |  | F_P2_ | | GAAGGTCGGAGTCAACGGATTCAAAACATCCAAATTTGCCCATGC |
|  |  | R | | GGGTACACATATACAAGGGTGACA |
| 21QP394 | chrE10 65159470 | F_P1_ | | GAAGGTGACCAAGTTCATGCTCTTATGTTGGACCCGTGCAC |
|  |  | F_P2_ | | GAAGGTCGGAGTCAACGGATTCTTATGTTGGACCCGTGCAT |
|  |  | R | | TTCTAGCTCCTCCATAAATCTGCA |
| 21QP400 | chrE10 65706508 | F_P1_ | | GAAGGTGACCAAGTTCATGCTGTGCGGGTGTCACTCATAGC |
|  |  | F_P2_ | | GAAGGTCGGAGTCAACGGATTGTGCGGGTGTCACTCATAGT |
|  |  | R | | AAGCGCAAAATAATGCCAAAAACT |
| 21QP403 | chrE10 65602455 | F_P1_ | | GAAGGTGACCAAGTTCATGCTTGTTGTTGGTTGATTGACAATGTA |
|  |  | F_P2_ | | GAAGGTCGGAGTCAACGGATTTGTTGTTGGTTGATTGACAATGTG |
|  |  | R | | GGTGCGAATGAACTCAATAACACT |
| 21QP406 | chrE10 65500204 | F_P1_ | | GAAGGTGACCAAGTTCATGCTAAGAATGCAATCTCTATCCTTTCAG |
|  |  | F_P2_ | | GAAGGTCGGAGTCAACGGATTAAGAATGCAATCTCTATCCTTTCAA |
|  |  | R | | GAAAGCATCCAACTTTAATGCCCT |
| 21QP409 | chrE10 65405924 | F_P1_ | | GAAGGTGACCAAGTTCATGCTGCTCACAAACAAAAATTGGATTAGCC |
|  |  | F_P2_ | | GAAGGTCGGAGTCAACGGATTGCTCACAAACAAAAATTGGATTAGCT |
|  |  | R | | CTCGTACAAGCTTCAGACTTTGAC |
| 21QP412 | chrE10 65744157 | F_P1_ | | GAAGGTGACCAAGTTCATGCTACATTTGATAAATTAGTTTGACTTCCAACT |
|  |  | F_P2_ | | GAAGGTCGGAGTCAACGGATTACATTTGATAAATTAGTTTGACTTCCAACA |
|  |  | R | | ACATGTAGGGACAGCATTCACTAG |
| 21QP415 | chrE10 64816418 | F_P1_ | | GAAGGTGACCAAGTTCATGCTGCATTATGCTTGTAGAAACTTCTCGTAT |
|  |  | F_P2_ | | GAAGGTCGGAGTCAACGGATTGCATTATGCTTGTAGAAACTTCTCGTAC |
|  |  | R | | TTTATGAAAGCACACAAGCCACAA |
| 21QP418 | chrE10 64534686 | F_P1_ | | GAAGGTGACCAAGTTCATGCTTCATCTTCTGAAGTTGGTTGTTTC |
|  |  | F_P2_ | | GAAGGTCGGAGTCAACGGATTTCATCTTCTGAAGTTGGTTGTTTA |
|  |  | R | | CCCTCTTTCTTCAGAAAATGCGTT |
| 21QP421 | chrE10 64552099 | F_P1_ | | GAAGGTGACCAAGTTCATGCTCCCACAACCAACCTCCCAAT |
|  |  | F_P2_ | | GAAGGTCGGAGTCAACGGATTCCCACAACCAACCTCCCAAG |
|  |  | R | | GCATCTAAAAGAGCCATCCTAGGA |
| 21QP424 | chrE10 77567463 | F_P1_ | | GAAGGTGACCAAGTTCATGCTTCCTCTGCAATCAATAGACAACAA |
|  |  | F_P2_ | | GAAGGTCGGAGTCAACGGATTTCCTCTGCAATCAATAGACAACAT |
|  |  | R | | GCCAGCACTATCAGCAAATAAGAG |
| 21QP427 | chrE10 77484460 | F_P1_ | | GAAGGTGACCAAGTTCATGCTCTGTGGCCGTTGTTAATTTTTCTG |
|  |  | F_P2_ | | GAAGGTCGGAGTCAACGGATTCTGTGGCCGTTGTTAATTTTTCTA |
|  |  | R | | TGGTCAAACCACTAACTAGTCCTG |
| 21QP438 | chrE10 65706328 | F_P1_ | | GAAGGTGACCAAGTTCATGCTGTGCGGGTGTCACTCATAGC |
|  |  | F_P2_ | | GAAGGTCGGAGTCAACGGATTGTGCGGGTGTCACTCATAGT |
|  |  | R | | AAGCGCAAAATAATGCCAAAAACT |
| 21QP444 | chrE10 67220893 | F_P1_ | | GAAGGTGACCAAGTTCATGCTCCAAACAAACACATTAATTTAGGCATG |
|  |  | F_P2_ | | GAAGGTCGGAGTCAACGGATTCCAAACAAACACATTAATTTAGGCATA |
|  |  | R | | AGAAGAGAGAAATGCAACCACTCA |
| 21QP450 | chrE10 78187579 | F_P1_ | | GAAGGTGACCAAGTTCATGCTACATTTAGTTGAATAGATTTTGTAAAAGCT |
|  |  | F_P2_ | | GAAGGTCGGAGTCAACGGATTACATTTAGTTGAATAGATTTTGTAAAAGCA |
|  |  | R | | CGGGTCGACTTGGATATATCTCAG |
| 22QP77 | chrE10 9989994 | F_P1_ | | GAAGGTGACCAAGTTCATGCTGGATCGTTTGACCAAATCTGCCT |
|  |  | F_P2_ | | GAAGGTCGGAGTCAACGGATTGGATCGTTTGACCAAATCTGCCC |
|  |  | R | | GAATCCGTGAAGCCGAATTATCTG |
| 22QP80 | chrE10 30022730 | F_P1_ | | GAAGGTGACCAAGTTCATGCTGCATTTAAACATGACTTATAGAGTAACTTGTAC |
|  |  | F_P2_ | | GAAGGTCGGAGTCAACGGATTGCATTTAAACATGACTTATAGAGTAACTTGTAA |
|  |  | R | | GACATTAGGTGATTGCTGTGCTAC |
| 22QP83 | chrE10 50008086 | F_P1_ | | GAAGGTGACCAAGTTCATGCTAATACCTAGCAGTCGAATTCCTTT |
|  |  | F_P2_ | | GAAGGTCGGAGTCAACGGATTAATACCTAGCAGTCGAATTCCTTC |
|  |  | R | | TATCATCTCATAGTTGCGCATTGC |
| 22QP86 | chrE10 62053354 | F_P1_ | | GAAGGTGACCAAGTTCATGCTACGGCCTATGGGTTGGGC |
|  |  | F_P2_ | | GAAGGTCGGAGTCAACGGATTACGGCCTATGGGTTGGGT |
|  |  | R | | TGTCGAGACACAAACCCTCTTATT |
| 22QP92 | chrE10 65151249 | F_P1_ | | GAAGGTGACCAAGTTCATGCTGAAGGGGGTAGGGGAAGGG |
|  |  | F_P2_ | | GAAGGTCGGAGTCAACGGATTGAAGGGGGTAGGGGAAGGA |
|  |  | R | | TCAAATTGTAATAGGCTAAATCAACTTTCT |
| 22QP95 | chrE10 66108325 | F_P1_ | | GAAGGTGACCAAGTTCATGCTCATCAGCACCTACACTTATCCCAG |
|  |  | F_P2_ | | GAAGGTCGGAGTCAACGGATTCATCAGCACCTACACTTATCCCAA |
|  |  | R | | GACGATGCTTGGGTGATAGTAGAT |
| 22QP98 | chrE10 68009252 | F_P1_ | | GAAGGTGACCAAGTTCATGCTTTGCCACGACATTACACCTTCA |
|  |  | F_P2_ | | GAAGGTCGGAGTCAACGGATTTTGCCACGACATTACACCTTCG |
|  |  | R | | CCAAATGGACCGGAATCTTTTCTT |
| 22QP101 | chrE10 70017068 | F_P1_ | | GAAGGTGACCAAGTTCATGCTCTGAAAGCAGTCAACCCGGC |
|  |  | F_P2_ | | GAAGGTCGGAGTCAACGGATTCTGAAAGCAGTCAACCCGGT |
|  |  | R | | TTTAAAGGCAATGACAGCGGATAC |
| 22QP104 | chrE10 72047818 | F_P1_ | | GAAGGTGACCAAGTTCATGCTAGGATGCAAGCAGACAGTAGAATG |
|  |  | F_P2_ | | GAAGGTCGGAGTCAACGGATTAGGATGCAAGCAGACAGTAGAATT |
|  |  | R | | AAATGGCCCAAAATATCGAGGTTG |
| 22QP107 | chrE10 73818464 | F_P1_ | | GAAGGTGACCAAGTTCATGCTGCAGCGAATGTTCCACCGAT |
|  |  | F_P2_ | | GAAGGTCGGAGTCAACGGATTGCAGCGAATGTTCCACCGAC |
|  |  | R | | CTGACGTATAAACAAGAGCTGAGC |
| 22QP110 | chrE10 74526778 | F_P1_ | | GAAGGTGACCAAGTTCATGCTCAGGAGTTGTCGGGCTATCTG |
|  |  | F_P2_ | | GAAGGTCGGAGTCAACGGATTCAGGAGTTGTCGGGCTATCTC |
|  |  | R | | GTGCTCAGATGAAACCAGACATTG |
| 22QP113 | chrE10 76470103 | F_P1_ | | GAAGGTGACCAAGTTCATGCTCGGACCCTTACGTGATGAGCTA |
|  |  | F_P2_ | | GAAGGTCGGAGTCAACGGATTCGGACCCTTACGTGATGAGCTT |
|  |  | R | | ACTGACGTTTTGGGACTTTTAAGA |
| 22QP116 | chrE10 80001359 | F_P1_ | | GAAGGTGACCAAGTTCATGCTAGTTAAAGTCATTGGGTCTTTTGATTATTT |
|  |  | F_P2_ | | GAAGGTCGGAGTCAACGGATTAGTTAAAGTCATTGGGTCTTTTGATTATTG |
|  |  | R | | TGGATATATACCTTCATTTAAACTCTCTGT |
| 22QP119 | chrE10 82037970 | F_P1_ | | GAAGGTGACCAAGTTCATGCTACCTCTGCAGAAGTAAAGACAATG |
|  |  | F_P2_ | | GAAGGTCGGAGTCAACGGATTACCTCTGCAGAAGTAAAGACAATA |
|  |  | R | | GAGTGTCATGACCTGAAAAGCATT |
| 22QP143 | chrE10 2113945 | F_P1_ | | GAAGGTGACCAAGTTCATGCTTATGCTATGGGCTGAAGAGGAAGC |
|  |  | F_P2_ | | GAAGGTCGGAGTCAACGGATTTATGCTATGGGCTGAAGAGGAAGT |
|  |  | R | | GCAATCTTACGCGCTATTTACTCA |
| 22QP146 | chrE10 50023118 | F_P1_ | | GAAGGTGACCAAGTTCATGCTACCTGTCCTTGACCCCTAAAAAC |
|  |  | F_P2_ | | GAAGGTCGGAGTCAACGGATTACCTGTCCTTGACCCCTAAAAAT |
|  |  | R | | GGGCGAATGTTATAAGAATGTGGG |
| 22QP149 | chrE10 62102649 | F_P1_ | | GAAGGTGACCAAGTTCATGCTCCAACTTGTTGTCCAGATTGACTG |
|  |  | F_P2_ | | GAAGGTCGGAGTCAACGGATTCCAACTTGTTGTCCAGATTGACTA |
|  |  | R | | GAGGGCACATGAATGGTATGGTAT |
| 22QP152 | chrE10 64355217 | F_P1_ | | GAAGGTGACCAAGTTCATGCTGTACAATTCTCTTTCAACATACAAAATTATTTA |
|  |  | F_P2_ | | GAAGGTCGGAGTCAACGGATTGTACAATTCTCTTTCAACATACAAAATTATTTG |
|  |  | R | | TAGCTCGCTACTAGTGCTTCATTC |
| 22QP158 | chrE10 82038029 | F_P1_ | | GAAGGTGACCAAGTTCATGCTGTCATGACACTCTTACGACAATAGAT |
|  |  | F_P2_ | | GAAGGTCGGAGTCAACGGATTGTCATGACACTCTTACGACAATAGAA |
|  |  | R | | CTTGTTCTCTCTCGTTCGAGGTTA |
| 22QP446 | chrE10 64355217 | F_P1_ | | GAAGGTGACCAAGTTCATGCTGTACAATTCTCTTTCAACATACAAAATTATTTA |
|  |  | F_P2_ | | GAAGGTCGGAGTCAACGGATTGTACAATTCTCTTTCAACATACAAAATTATTTG |
|  |  | R | | TAGCTCGCTACTAGTGCTTCATTC |
| 22QP449 | chrE10 62355793 | F_P1_ | | GAAGGTGACCAAGTTCATGCTAGATATTACATTCTTCATTTTGAGATGCAA |
|  |  | F_P2_ | | GAAGGTCGGAGTCAACGGATTAGATATTACATTCTTCATTTTGAGATGCAG |
|  |  | R | | TTGTCTAATCACAACGCAAGAACC |
| 22QP452 | chrE10 60058927 | F_P1_ | | GAAGGTGACCAAGTTCATGCTAGAAGTTTCATTGTGATTTCCCTTC |
|  |  | F_P2_ | | GAAGGTCGGAGTCAACGGATTAGAAGTTTCATTGTGATTTCCCTTT |
|  |  | R | | ATAGTGATAGGATTGCTCCACCAC |
| 22QP455 | chrE10 57907207 | F_P1_ | | GAAGGTGACCAAGTTCATGCTGCTGCGAAATCCCATGTAGCT |
|  |  | F_P2_ | | GAAGGTCGGAGTCAACGGATTGCTGCGAAATCCCATGTAGCA |
|  |  | R | | GGGTAGTTGGAGAGAACTTGACAT |
| 22QP458 | chrE10 55878168 | F_P1_ | | GAAGGTGACCAAGTTCATGCTCCTACAAATGCCATCAACTCTTATACT |
|  |  | F_P2_ | | GAAGGTCGGAGTCAACGGATTCCTACAAATGCCATCAACTCTTATACC |
|  |  | R | | CTCTTTAGGGCTAAAACCAGGGAT |
| 22QP461 | chrE10 66346011 | F_P1_ | | GAAGGTGACCAAGTTCATGCTATTTTACGAGGTGGATGCATGTGT |
|  |  | F_P2_ | | GAAGGTCGGAGTCAACGGATTATTTTACGAGGTGGATGCATGTGC |
|  |  | R | | GTCTGGCAATAAACGTGCTGAAAT |
| 22QP464 | chrE10 68142176 | F_P1_ | | GAAGGTGACCAAGTTCATGCTTTGATTCTCGTGAATGCAACAGAT |
|  |  | F_P2_ | | GAAGGTCGGAGTCAACGGATTTTGATTCTCGTGAATGCAACAGAA |
|  |  | R | | CTGAGTAAAACCTTCGCTTCCTTC |
| 22QP491 | chrE10 74114513 | F_P1_ | | GAAGGTGACCAAGTTCATGCTCACTAGGTATAGGTGTAGGGGGTAT |
|  |  | F_P2_ | | GAAGGTCGGAGTCAACGGATTCACTAGGTATAGGTGTAGGGGGTAC |
|  |  | R | | TTCCCATTTCTGACAAGGCAAATC |
| 22QP500 | chrE10 69711802 | F_P1_ | | GAAGGTGACCAAGTTCATGCTGAATTTCAACGTGGACATTGAGCA |
|  |  | F_P2_ | | GAAGGTCGGAGTCAACGGATTGAATTTCAACGTGGACATTGAGCG |
|  |  | R | | AGGTTTCCCCAACATGCAATAATG |
| 23QP187 | chrE10 64816418 | F_P1_ | | GAAGGTGACCAAGTTCATGCTGCATTATGCTTGTAGAAACTTCTCGTAT |
|  |  | F_P2_ | | GAAGGTCGGAGTCAACGGATTGCATTATGCTTGTAGAAACTTCTCGTAC |
|  |  | R | | TTTATGAAAGCACACAAGCCACAA |
| 23QP190 | chrE10 65495705 | F_P1_ | | GAAGGTGACCAAGTTCATGCTGACGAGGGAGGAACATTTTTCAGT |
|  |  | F_P2_ | | GAAGGTCGGAGTCAACGGATTGACGAGGGAGGAACATTTTTCAGC |
|  |  | R | | CCGCCGCAGAATAATGAACTAGTA |
| 23QP193 | chrE10 68251934 | F_P1_ | | GAAGGTGACCAAGTTCATGCTTCTTCTGCTAACTATCCATAAACAAAC |
|  |  | F_P2_ | | GAAGGTCGGAGTCAACGGATTTCTTCTGCTAACTATCCATAAACAAAT |
|  |  | R | | TAACCGATCACAACTTGCTTAGGA |
| 23QP196 | chrE10 69369046 | F_P1_ | | GAAGGTGACCAAGTTCATGCTTTATTATCGCATCAAAAATTACAGGAAA |
|  |  | F_P2_ | | GAAGGTCGGAGTCAACGGATTTTATTATCGCATCAAAAATTACAGGAAG |
|  |  | R | | ATTTTCAGTGTTCGAACTCAACCC |
| 23QP199 | chrE10 70017068 | F_P1_ | | GAAGGTGACCAAGTTCATGCTCTGAAAGCAGTCAACCCGGC |
|  |  | F_P2_ | | GAAGGTCGGAGTCAACGGATTCTGAAAGCAGTCAACCCGGT |
|  |  | R | | TTTAAAGGCAATGACAGCGGATAC |
| 23QP202 | chrE10 71249498 | F_P1_ | | GAAGGTGACCAAGTTCATGCTTCACAGTTAACTCAAAAACATTGCTA |
|  |  | F_P2_ | | GAAGGTCGGAGTCAACGGATTTCACAGTTAACTCAAAAACATTGCTT |
|  |  | R | | GTGGCATTTGAAGACCTTCTTTGA |
| 23QP205 | chrE10 72177387 | F_P1_ | | GAAGGTGACCAAGTTCATGCTCCATCATTCATCAAACTCCATTCTTG |
|  |  | F_P2_ | | GAAGGTCGGAGTCAACGGATTCCATCATTCATCAAACTCCATTCTTA |
|  |  | R | | TTGGAGGGGTTGCTATTACAAGTT |
| 23QP459 | chrE10 76636742 | F_P1_ | | GAAGGTGACCAAGTTCATGCTAGAATAGAACGTTTGTAGACCTGA |
|  |  | F_P2_ | | GAAGGTCGGAGTCAACGGATTAGAATAGAACGTTTGTAGACCTGG |
|  |  | R | | TGCTGGATTTACTTGTGGCAATTT |
| 23QP518 | chrE10 79993845 | F_P1_ | | GAAGGTGACCAAGTTCATGCTTCTGCATTTGTGTTTCTTCGTCG |
|  |  | F_P2_ | | GAAGGTCGGAGTCAACGGATTTCTGCATTTGTGTTTCTTCGTCA |
|  |  | R | | ACGACGACGAATAATACAGACGTA |
| 23QP566 | chrE10 77988369 | F_P1_ | | GAAGGTGACCAAGTTCATGCTTTATGAAATCAACCCGCAAATAAAC |
|  |  | F_P2_ | | GAAGGTCGGAGTCAACGGATTTTATGAAATCAACCCGCAAATAAAT |
|  |  | R | | TGACTTTTGAGTCAGCATAGCAAC |
| 23QP572 | chrE10 64357565 | F_P1_ | | GAAGGTGACCAAGTTCATGCTAGTTACAAGATTAAGGGATATCTGAATG |
|  |  | F_P2_ | | GAAGGTCGGAGTCAACGGATTAGTTACAAGATTAAGGGATATCTGAATA |
|  |  | R | | AGGCAAAAAGATAGGCTTGAGGTA |
| 23QP578 | chrE10 76483336 | F_P1_ | | GAAGGTGACCAAGTTCATGCTCAATGATGACGCTCACAATTCATT |
|  |  | F_P2_ | | GAAGGTCGGAGTCAACGGATTCAATGATGACGCTCACAATTCATC |
|  |  | R | | GCAATAAGTGACCATCTACACAAA |
| 23QP581 | chrE10 76481733 | F_P1_ | | GAAGGTGACCAAGTTCATGCTTTTTCCACTGTCAATCATTGAAAATC |
|  |  | F_P2_ | | GAAGGTCGGAGTCAACGGATTTTTTCCACTGTCAATCATTGAAAATT |
|  |  | R | | TATGTTTTCTAGTGGGCTGGTTCA |
| 23QP602 | chrE10 76569359 | F_P1_ | | GAAGGTGACCAAGTTCATGCTGCAGTTCATTAGCATAGATTTTCTTCTGA |
|  |  | F_P2_ | | GAAGGTCGGAGTCAACGGATTGCAGTTCATTAGCATAGATTTTCTTCTGT |
|  |  | R | | CACTCCAATCTGAGACTTAGCCTT |
| 23QP953 | chrE10 76497112 | F_P1_ | | GAAGGTGACCAAGTTCATGCTTCTCTGAATGGTTGTTATGTCGAT |
|  |  | F_P2_ | | GAAGGTCGGAGTCAACGGATTTCTCTGAATGGTTGTTATGTCGAC |
|  |  | R | | CAAGAGAGGACTATAGGCTTTCCA |
| 23QP956 | chrE10 76510458 | F_P1_ | | GAAGGTGACCAAGTTCATGCTAATGTTATTCCACCACTAAATATAAGTAGTA |
|  |  | F_P2_ | | GAAGGTCGGAGTCAACGGATTAATGTTATTCCACCACTAAATATAAGTAGTG |
|  |  | R | | ACTGCGATTTTAGTGAGTTGTCAA |
| 23QP959 | chrE10 76511218 | F_P1_ | | GAAGGTGACCAAGTTCATGCTACTAACTTTGAACATAAGTGACAATCC |
|  |  | F_P2_ | | GAAGGTCGGAGTCAACGGATTACTAACTTTGAACATAAGTGACAATCT |
|  |  | R | | TTTCATAATTGTAGCCAAGCAACA |
| **Primers used for qRT-PCR** | | | | |
| Primer Name | F/R | Primer Sequence | | |
| *SmMYB113* | F | TACGTCTGTGCGAGTGAGGA | | |
|  | R | TCCGAAGCAAAGTCACCTCT | | |
| *SmCHS* | F | TCCATTACCAGAAGTCGAAAG | | |
|  | R | | TTCCAATCAGAAATGCCTAA | |
| *SmF3’5’H* | F | | ACCCTATGGACCTCGTTGGA | |
|  | R | GCGAACGTCAACATATCGGC | | |
| *SmCyclophilin* | F | GCGCCAAATTCAAGGACGAGAACT | | |
|  | R | ACAGCCTCGGCCTTCTTAATCACA | | |
| 23QP711 | F | CTGCTTACCTATACACCCCGTC | | |
|  | R | TAGAGGTATCCAGGGGTCGAAG | | |
| 23QP713 | F | ATCAATGAACCACCTTCGACGA | | |
|  | R | GTACGGAACAGCGGAATCAAAA | | |
| 23QP715 | F | TGACTTTTAGATCTGTGCATTGCA | | |
|  | R | TTTCTTGACCCCCTTTCCACC | | |
| *SlActin* | F | GGGATGGAGAAGTTTGGTGGTGG | | |
|  | R | CTTCGACCAAGGGATGGTGTAGC | | |
| **Primers used for RU analysis** | | | | |
| Primer Name | F/R | Primer Sequence | | |
| 23QP925 | F | TGCTTACCTATACACCCCGTCTA | | |
|  | R | TGCAATGCACAGATCTAAAAGTCA | | |
| 23QP723 | F | CCAGGCTATCATCATATAGGGTGTT | | |
|  | R | AAAGTGAAATAATACGAAAAACCCCAT | | |
| **Primers used for plasmid construction** | | | | |
| Primer Name | F/R | Primer Sequence | | |
| *SmMYB113* | F | CCATGATTACGAATTCCCTCGGGTCTCTTTCTA | | |
|  | R | ATGGTCTAGAGGATCCATCAAGTAGATTCCATAAATCAAT | | |
