# Supplementary figures and images for: A 725-bp quadruple repeat in the promoter of SmMYB113 is associated with light-independent anthocyanin regulation in eggplant
Source: Hortic Res. 2025 Nov 21;13(3):uhaf319. doi: 10.1093/hr/uhaf319 (PMC12962852; doi:10.1093/hr/uhaf319)

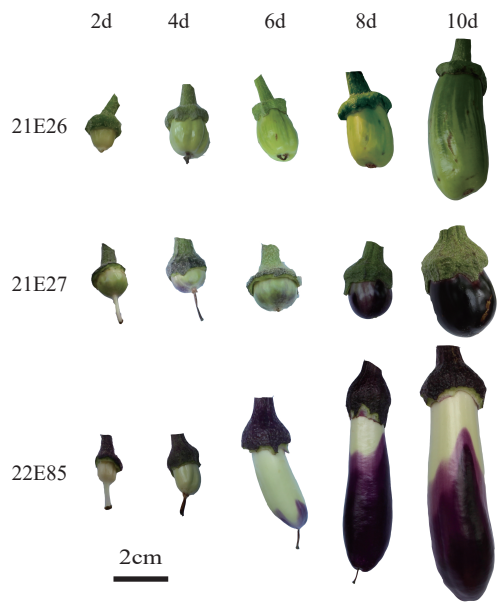

Supplement: Web_Material_uhaf319 [file web_material_uhaf319.zip › Figure S1.pdf]

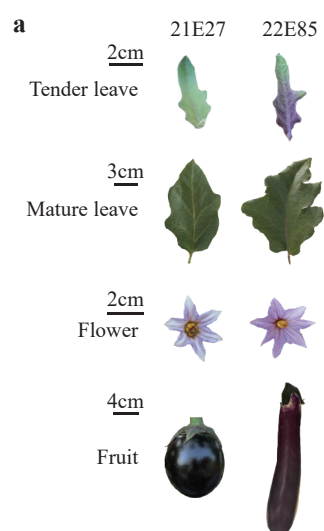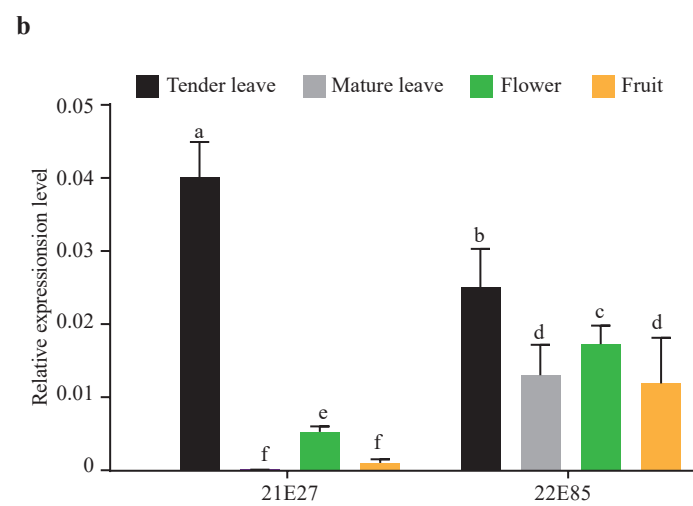

Supplement: Web_Material_uhaf319 [file web_material_uhaf319.zip › Figure S10.pdf]

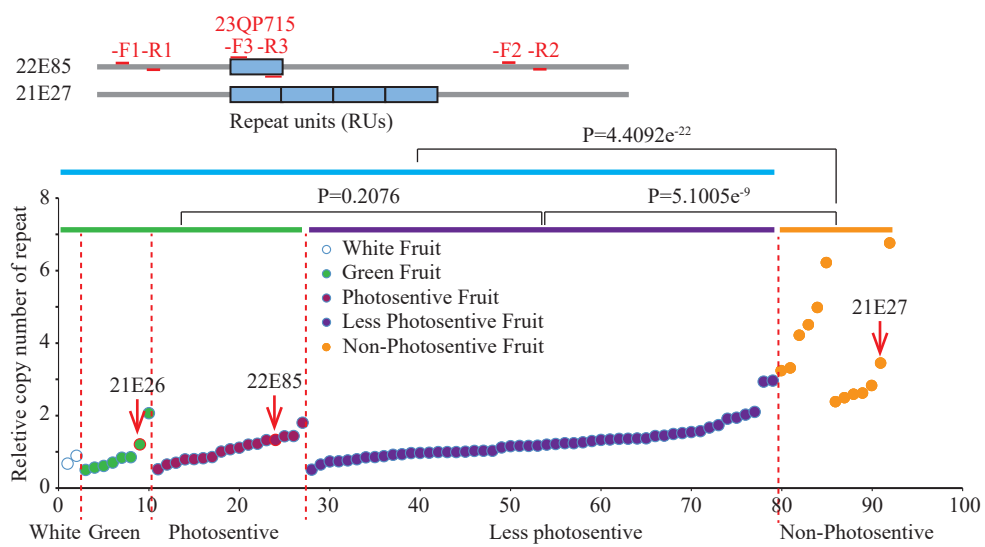

Supplement: Web_Material_uhaf319 [file web_material_uhaf319.zip › Figure S11.pdf]

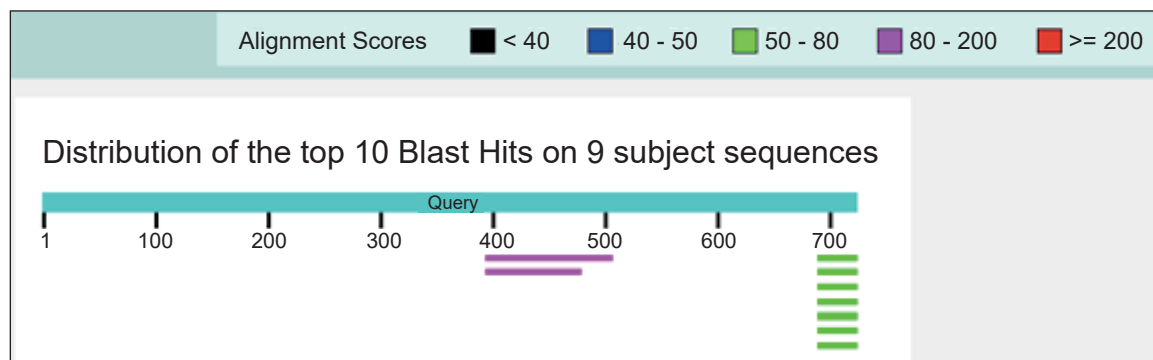

Supplement: Web_Material_uhaf319 [file web_material_uhaf319.zip › Figure S12.pdf]

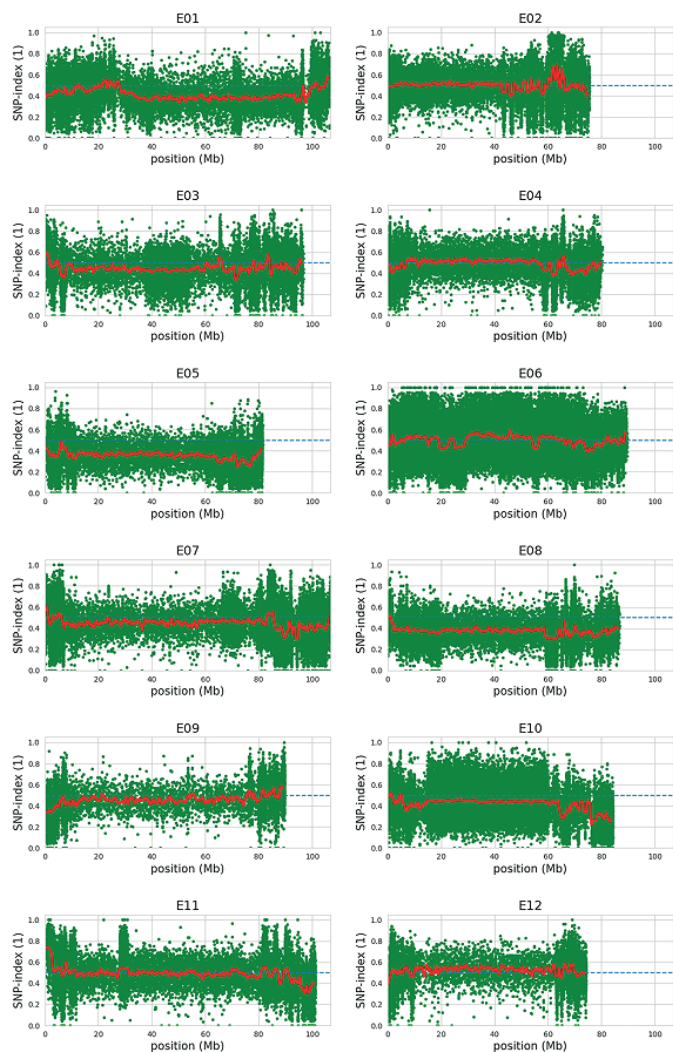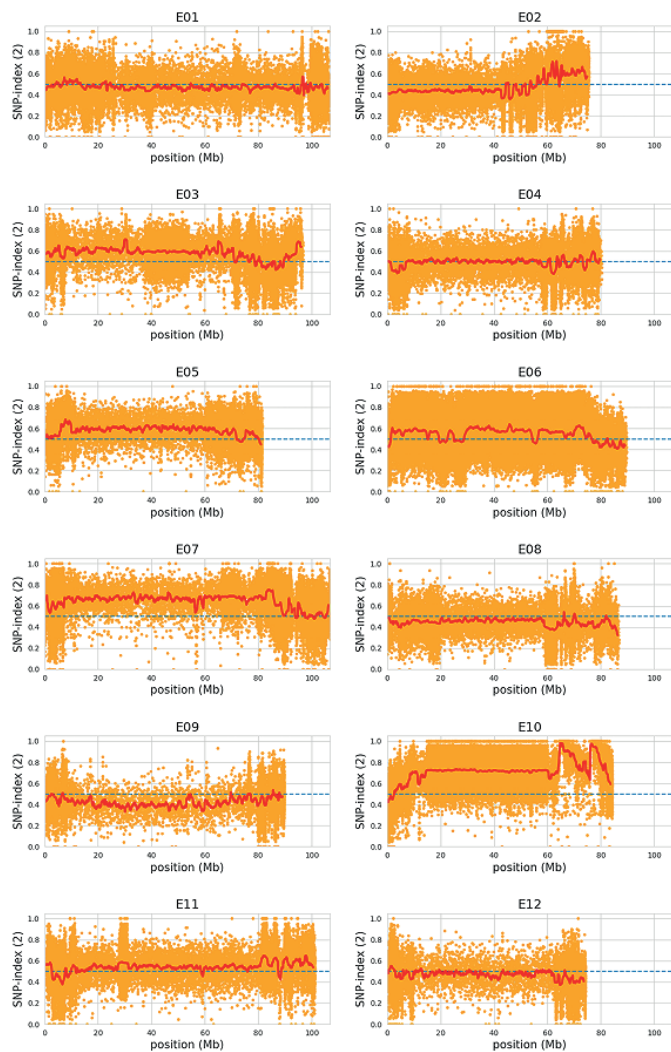

Supplement: Web_Material_uhaf319 [file web_material_uhaf319.zip › Figure S3.pdf]

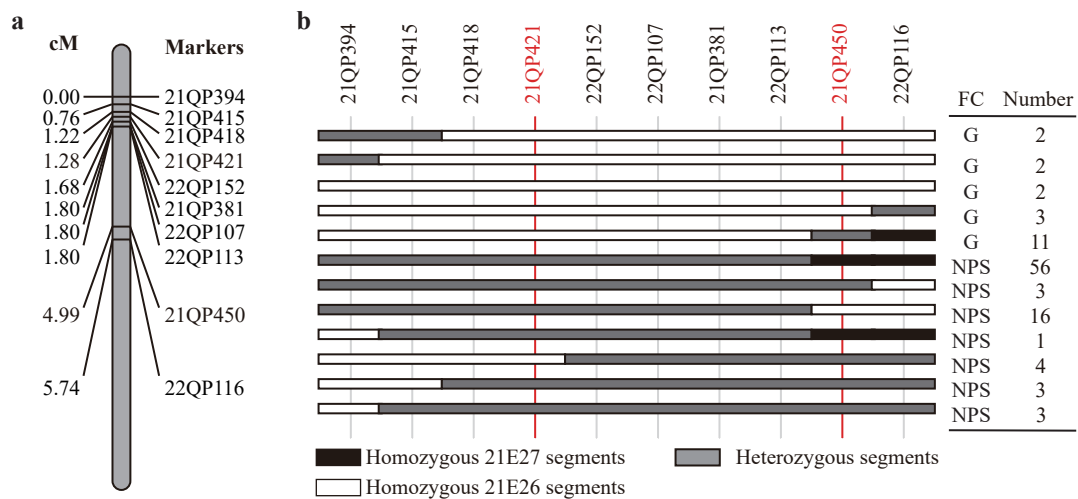

Supplement: Web_Material_uhaf319 [file web_material_uhaf319.zip › Figure S4.pdf]

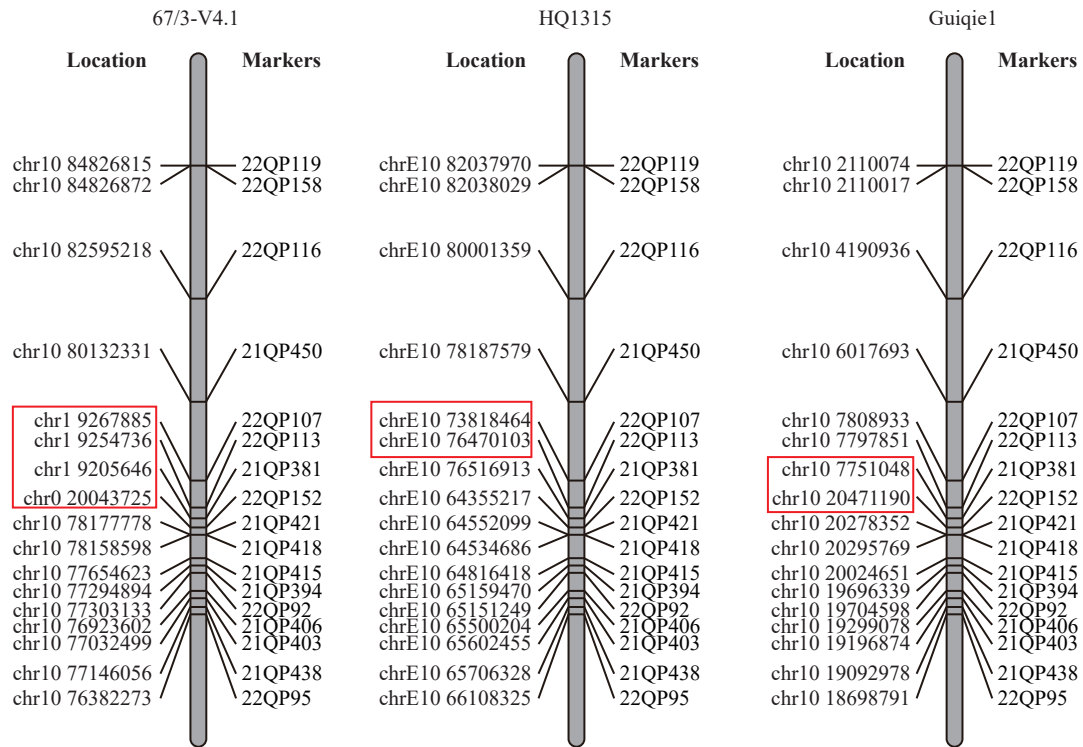

Supplement: Web_Material_uhaf319 [file web_material_uhaf319.zip › Figure S5.pdf]

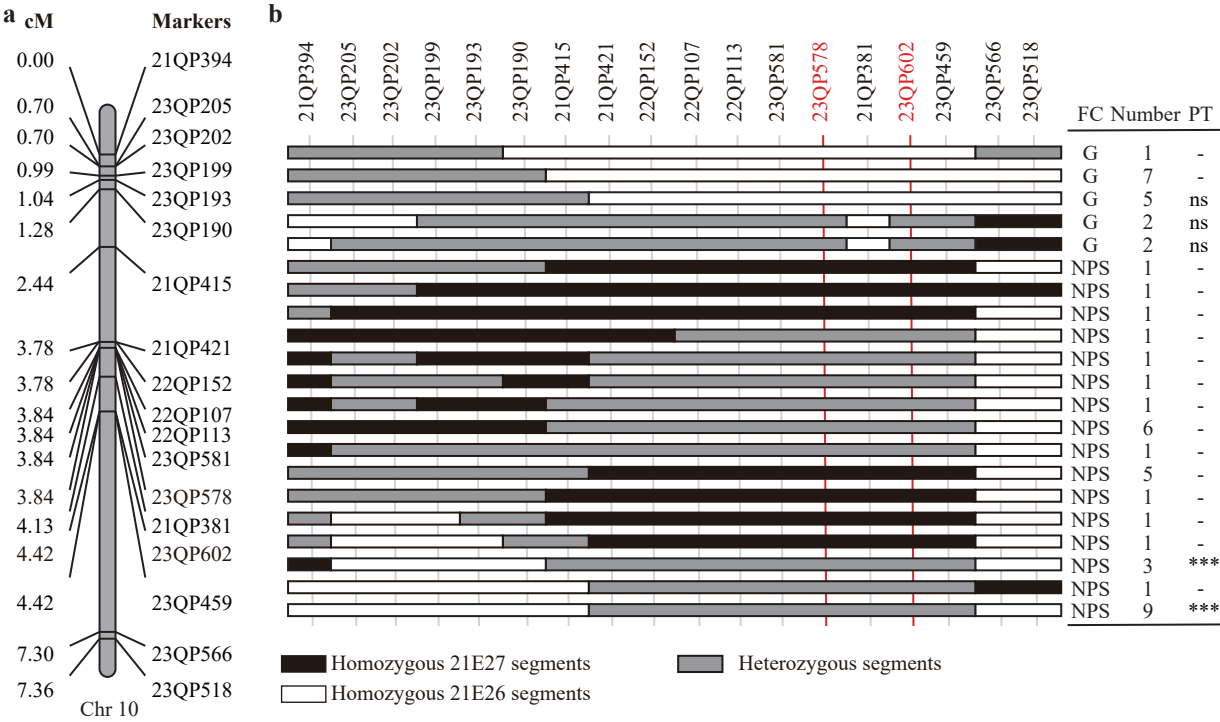

Supplement: Web_Material_uhaf319 [file web_material_uhaf319.zip › Figure S6.pdf]

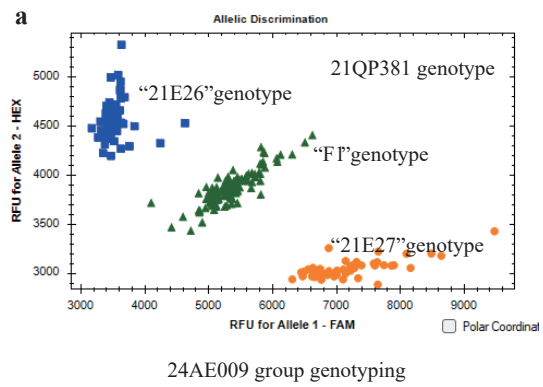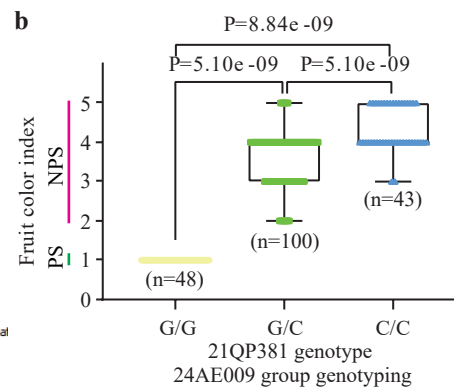

Supplement: Web_Material_uhaf319 [file web_material_uhaf319.zip › Figure S7.pdf]
